# Age‐associated vascular inflammation promotes monocytosis during atherogenesis

**DOI:** 10.1111/acel.12488

**Published:** 2016-05-02

**Authors:** Wei Du, Christine Wong, Yang Song, Hua Shen, Daniel Mori, Noemi Rotllan, Nathan Price, Anca D. Dobrian, Hailong Meng, Steven H. Kleinstein, Carlos Fernandez‐Hernando, Daniel R. Goldstein

**Affiliations:** ^1^ Department of Internal MedicineYale School of MedicineNew HavenCTUSA; ^2^ Department of ImmunobiologyYale School of MedicineNew HavenCTUSA; ^3^ Section of Comparative MedicineYale School of MedicineNew HavenCTUSA; ^4^ Department of Physiological SciencesEastern Virginia Medical SchoolNorfolkVAUSA; ^5^ Department of PathologyYale School of MedicineNew HavenCTUSA; ^6^ Interdepartmental Program in Computational Biology and BioinformaticsYale UniversityNew HavenCTUSA

**Keywords:** aging, monocyte, vasculature, inflammation and atherosclerosis

## Abstract

Aging leads to a proinflammatory state within the vasculature without disease, yet whether this inflammatory state occurs during atherogenesis remains unclear. Here, we examined how aging impacts atherosclerosis using *Ldlr* ^*−/−*^ mice, an established murine model of atherosclerosis. We found that aged atherosclerotic *Ldlr* ^*−/−*^ mice exhibited enhanced atherogenesis within the aorta. Aging also led to increased LDL levels, elevated blood pressure on a low‐fat diet, and insulin resistance after a high‐fat diet (HFD). On a HFD, aging increased a monocytosis in the peripheral blood and enhanced macrophage accumulation within the aorta. When we conducted bone marrow transplant experiments, we found that stromal factors contributed to age‐enhanced atherosclerosis. To delineate these stromal factors, we determined that the vasculature exhibited an age‐enhanced inflammatory response consisting of elevated production of CCL‐2, osteopontin, and IL‐6 during atherogenesis. In addition, *in vitro* cultures showed that aging enhanced the production of osteopontin by vascular smooth muscle cells. Functionally, aged atherosclerotic aortas displayed higher monocyte chemotaxis than young aortas. Hence, our study has revealed that aging induces metabolic dysfunction and enhances vascular inflammation to promote a peripheral monocytosis and macrophage accumulation within the atherosclerotic aorta.

## Introduction

Atherosclerosis in the aging population has well surpassed other age‐associated diseases such as susceptibility to infection, chronic lung disease, and cancer as a cause of morbidity and mortality in older people (Ferrari *et al*., 2003; Mozaffarian *et al*., 2015). The strongest independent risk factor for the development of atherosclerosis is aging. This risk is greater than the additive risk of hypertension, hypercholesterolemia, and genetics accrued over time (Ferrari *et al*., 2003). Despite the ongoing threat of atherosclerosis in older people, our understanding of the mechanisms by which aging enhances atherosclerosis remains unclear and under investigated especially in relevant experimental disease models (Collins *et al*., 2009; Wang & Bennett, 2012).

Aging may affect atherosclerosis through several mechanisms in hematopoietic cells, vascular cells, or both (Wang & Bennett, 2012; Gioscia‐Ryan *et al*., 2014). For example, aging induces cellular senescence, which leads to DNA damage and impaired antioxidant responses resulting in vascular inflammation that contributes to atherosclerosis (Collins *et al*., 2009; Wang & Bennett, 2012). In addition, studies in disease‐free animals have found that vascular aging induces oxidative stress in endothelial cells (Ungvari *et al*., 2010; Gano *et al*., 2014), and leads to medial vessel wall thickening, increased collagen, and extracellular matrix deposition (Wang *et al*., 2014). Endothelial cells (ECs) and vascular smooth muscle cells (VSMC) from disease‐free animals exhibit enhanced secretion of inflammatory mediators with aging (Spinetti *et al*., 2004; Donato *et al*., 2008; Ungvari *et al*., 2010; Csiszar *et al*., 2012; Gano *et al*., 2014; Wang *et al*., 2014). Hence, these studies indicate that vascular aging may predispose to diseases such as atherosclerosis, yet whether such age‐related vascular changes occur during atherosclerosis remains unclear.

Monocytes and their macrophage descendants are critical immune cells for atherosclerosis (Moore *et al*., 2013; Hilgendorf *et al*., 2015). Monocyte recruitment into aortas is critical during atherogenesis, whereas macrophage proliferation in the tissue enhances atherosclerotic lesion progression (Hilgendorf *et al*., 2015). Monocytes can be categorized into ‘inflammatory’ monocytes (defined by high surface expression of CD115, CCR2, and Gr1 and intermediate expression of Cx3CR1, the fractalkine receptor) and ‘patrolling’ monocytes (defined as CD115^hi^ CCR2^lo^ Cx3CR1^hi^ but Gr1^−ve^) (Geissmann *et al*., 2003). Inflammatory monocytes are typically the initial cells recruited into inflammatory sites of the aorta that develop atherosclerosis (Swirski *et al*., 2007). After recruitment, the cells engulf lipid particles, become ‘foam cell’ macrophages, and accumulate within atherosclerotic lesions (Woollard & Geissmann, 2010; Moore *et al*., 2013; Hilgendorf *et al*., 2015). It is not known, however, whether aging enhances monocyte intrinsic function or whether aging impacts monocytes indirectly via the vasculature during chronic inflammatory diseases such as atherosclerosis.

To examine vascular inflammation and monocyte function in the context of atherosclerosis and aging, we employed aged *Ldlr* ^*−/−*^ mice and found that aging promotes metabolic dysfunction notably hyperlipidemia, hypertension, and insulin resistance during atherogenesis. These changes with aging were accompanied by a peripheral blood monocytosis and an increased macrophage aortic accumulation. Bone marrow transplant studies uncovered that stromal factors contribute to age‐enhanced atherosclerosis. To uncover some of these stromal factors, we found that the aging atherosclerotic aortas exhibited enhanced production of inflammatory proteins including IL‐6 and the monocyte chemokines, CCL‐2 and osteopontin (OPN). We identified that VSMC as a contributory source of these inflammatory proteins. Finally, we discovered that aged atherosclerotic aorta, extrinsic to the monocyte, enhanced monocyte chemotaxis. Hence, our study has revealed that age‐associated tissue inflammation promotes monocyte chemotaxis during atherogenesis.

## Results

### Aging enhances atherosclerosis lesion size

**Figure 1:**
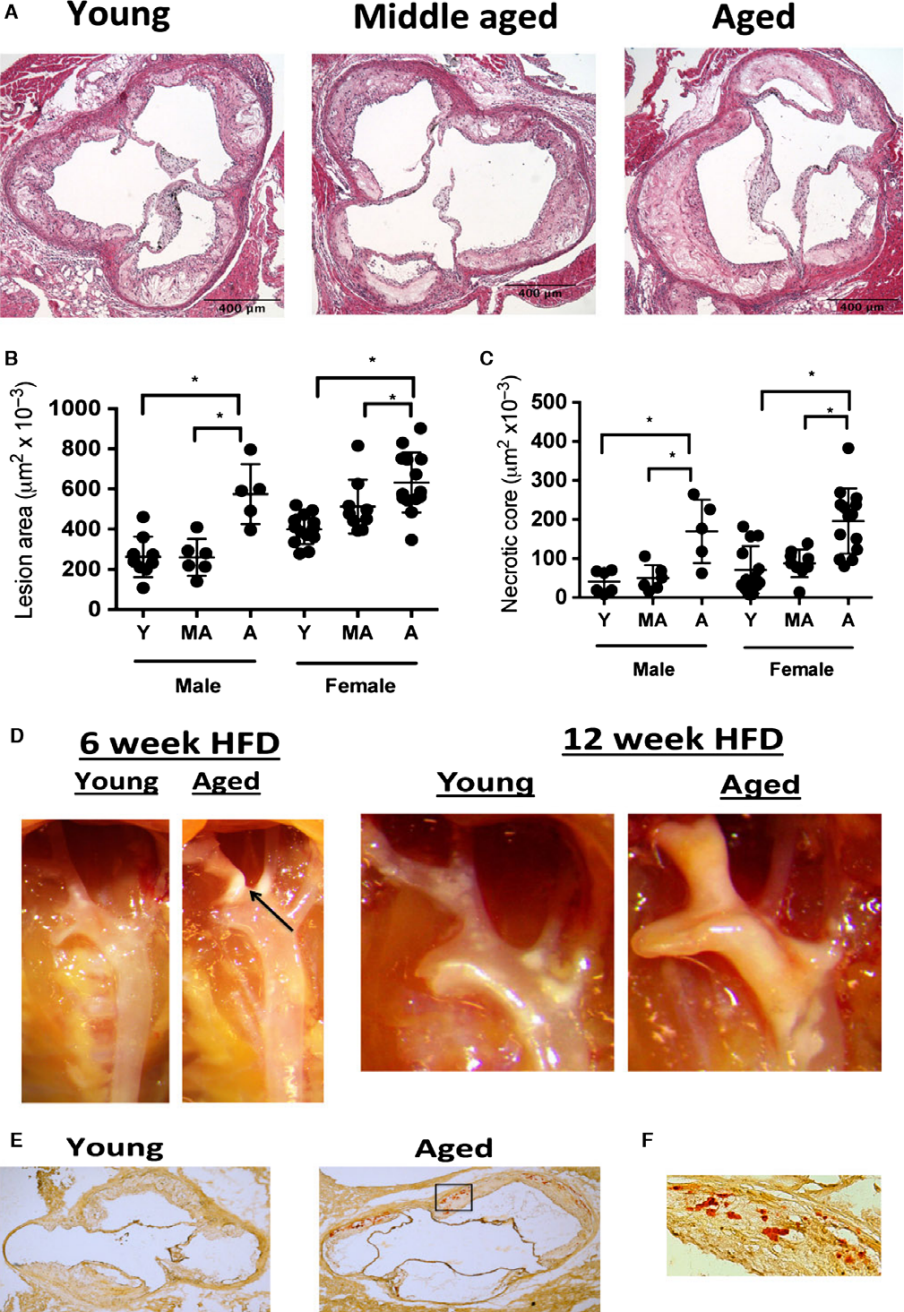
Aging enhances atherosclerosis during HFD. (A–B) Young (5 months of age), middle aged (11 months of age) and aged (15 months of age) male and female *Ldlr* ^−/−^ mice were maintained on a chow diet until 3 months prior to tissue harvest when they were switch to a HFD. At the end of the HFD feeding period, the aortic root was obtained and stained with H&E, and lesion size was enumerated. Representative images of male mice are shown in A, and quantification is shown B. *P < 0.01 (*t*‐test). (C) As per A, necrotic core lesion assessment based upon the area of acellular staining within the H&E images of the aortic root of the each of the experimental cohorts. *P < 0.01 (*t*‐test). (D) Representative photographs of the ascending aortic arch of aged or young male *Ldlr* ^−/−^ mice fed a diet for 6 or 12 weeks. Atherosclerotic plaque is seen as white, opaque material. (E) Young (5 months) and aged (15 months) male *Ldlr* ^−/−^ mice were fed a HFD for 3 months prior to tissue harvest and the lesions were stained with alizarin red to assess lesion calcification (red staining). A representative image of young and aged aortic root lesions are shown at 2×. *N* = 8 biological replicates/group. (F) Inset of the box of aged aortic root shown in G at 20×.

To examine how aging impacts atherosclerosis, we aged cohorts of *Ldlr* ^*−/−*^ mice, an established murine model of atherosclerosis (Getz & Reardon, 2006). We included young (5 months), middle aged (11 months), aged (15 months), and advanced aged (21 months) male *Ldlr* ^*−/−*^ mice that were maintained on a chow fed (i.e. low‐fat diet) except for the last 3 months prior to tissue harvest when the mice were switched to a high‐fat diet (HFD). We found that aged and advanced aged male mice exhibited increased atherosclerotic lesion size and a necrotic core area within the lesions as compared to young and middle aged male cohorts (Figs 1A–B, S1A‐C). Similar results were observed for young, middle aged and aged female *Ldlr* ^*−/−*^ mice although an advanced age group was not available (Fig. 1A–B). (Note: male mice were used for the rest of the study unless indicated.) Additionally, aged male *Ldlr* ^*−/−*^ mice exhibited increased plaque deposition within the aortic arch during the HFD period as compared to young male *Ldlr* ^*−/−*^ mice (Fig. 1F). Aged *Ldlr* ^*−/−*^ mice also exhibited higher lesion calcification than young *Ldlr* ^*−/−*^ mice (Fig. 1G–H). Wild‐type (WT) aged and young mice fed a high‐fat diet for 6 weeks did not elicit photographic evidence of atherosclerosis (Fig. S1D), confirming prior studies that WT mice fail to develop atherosclerosis during a HFD (Getz & Reardon, 2006).

**Figure 2:**
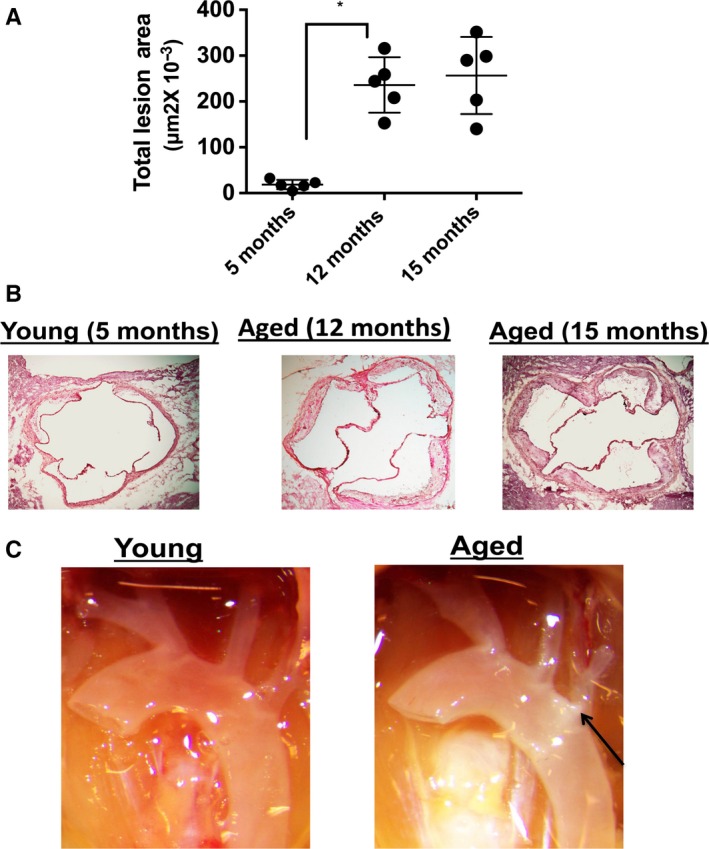
Aging enhances atherogenesis during chow (low‐fat) diet. (A) *Ldlr* ^−/−^ male mice were maintained on a chow diet for 5, 12 and 15 months and atherosclerotic lesion size was assessed within the aortic root. *P < 0.01 (*t*‐test). (B) Representative histological images of the aortic root of the differently aged groups in A. (C) Representative photographic images of the ascending aortic arch of young (5 months) and aged (15 months) mice. Arrow denotes white plaque.

We also maintained mice on a chow diet up to the ages of 5, 12, and 15 months without administering a HFD and assessed atherosclerosis disease development in the aortic root histologically and plaque deposition in the aortic arch photographically. Examination of the aortic root demonstrated that both 12‐ and 15‐month‐old *Ldlr* ^*−/−*^ mice exhibited atherosclerotic lesions, whereas 5‐month‐old *Ldlr* ^*−/−*^ mice exhibited minimal disease (Fig. 2A–C). In 15‐month‐old *Ldlr* ^*−/−*^ mice, there was photographic evidence of plaque deposition at branch points of the aortic arch, which was barely evident in 5‐month‐old *Ldlr* ^*−/−*^ mice (Fig. 2C), although chow‐fed mice (young or aged) exhibited considerably less evidence of plaque deposition within the ascending aortic arch than *Ldlr* ^*−/−*^ mice fed a HFD (see Fig. 1). Overall, these data indicate that aging associates with enhanced atherosclerosis in *Ldlr* ^*−/−*^ mice even without the administration of a HFD, which is typically given to young *Ldlr* ^*−/−*^ mice to enhance atherosclerotic disease development (Getz & Reardon, 2006).

### Aging increases LDL‐cholesterol on a low‐cholesterol diet

**Figure 3:**
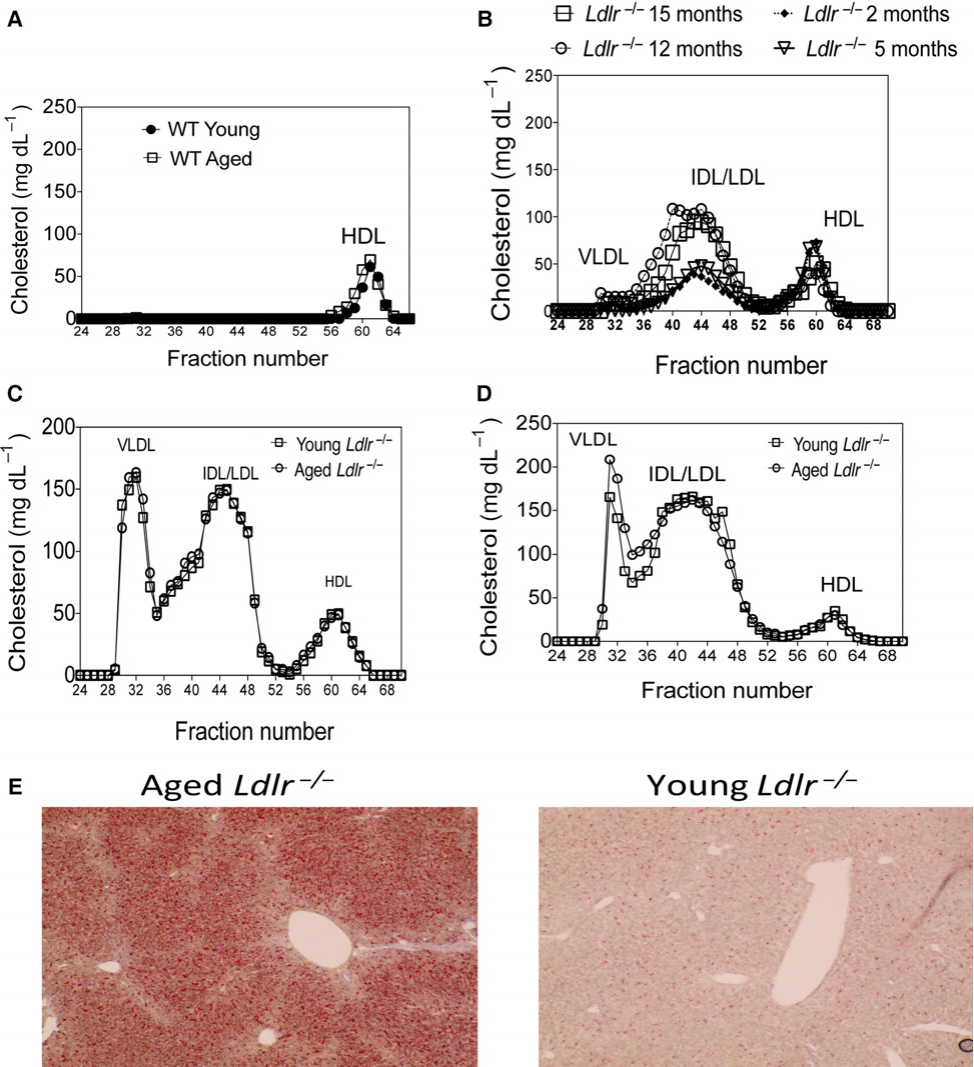
Aging *Ldlr* ^*−/−*^ mice exhibit increased lipid levels during chow diet. (A) WT young (2 months of age) and aged male (16 months of age) C57BL/6 mice were maintained on a chow diet and fasting plasma was obtained to assess lipid distribution via HPLC. *N* = 3–5/group. (B) *Ldlr* ^−/−^ male mice were maintained on a chow diet for 2, 5, 12 and 15 months and fasting plasma was obtained to assess lipid particle distribution via HPLC. *N* = 3–6 mice/group. (C–D) *Ldlr* ^−/−^ male young (2 months of age) and aged (12 months of age) mice were placed on a HFD for an additional 1 month (C) or 3 months (D) and fasting plasma was obtained for analysis via HPLC. *N* = 3–6 mice/group. (E) *Ldlr* ^−/−^ male mice were maintained on a chow diet for 2 or 12 months (*n* = 5/group), and then, their livers were obtained and stained for lipids by ORO staining. 10× magnification. Representative livers from aged and young mice are shown.

High‐performance liquid chromatography (HPLC) of fasting plasma of chow‐fed WT young (2 months of age) and aged (16 months of age) mice demonstrated that WT mice exhibit high‐density lipoprotein‐cholesterol (HDL) levels with no difference between young and aged mice (Fig. 3A). WT young and aged mice also failed to become hypercholesterolemic on a HFD in contrast to *Ldlr* ^*−/−*^ mice (Fig. S1E) consistent with prior studies that WT mice do not develop atherosclerosis even on a HFD (Getz & Reardon, 2006). Fasting serum low‐density lipoprotein‐cholesterol (LDL) levels increased in *Ldlr* ^*−/−*^ mice aged 12 and 15 months compared to *Ldlr* ^*−/−*^ mice aged 2 and 5 months (Fig. 3B). After 1 month of a HFD, 13 month aged and 3 month aged young *Ldlr* ^*−/−*^ mice exhibited a similar increase in VLDL and LDL levels (Fig. 3C), which was maintained after 3 months on the HFD (Fig. 3D). 12 month of age *Ldlr* ^*−/−*^ mice maintained on a chow diet also exhibited evidence of increased lipid deposition within the liver whereas 2 month of age *Ldlr* ^*−/−*^ mice did not (Fig. 3E). These findings indicate that as *Ldlr* ^*−/−*^ mice age on a chow diet they exhibit hyperlipidemia and lipid deposition within the liver.

### Aging induces metabolic dysfunction during a HFD and atherosclerosis

To characterize metabolic function in young and aged *Ldlr* ^*−/−*^ mice, we first measured their body weight. Aged *Ldlr* ^*−/−*^ mice exhibited increased body weight at 12 months of age prior to the HFD as compared to 2‐month‐old *Ldlr* ^*−/−*^ mice (Fig. S2A). This weight increase was maintained, but not enhanced, in aged *Ldlr* ^*−/−*^ mice during the course of the 3‐month HFD (Fig. S2A). But after 1 month of a HFD, aged *Ldlr* ^*−/−*^ mice exhibited increased adiposity of both subcutaneous and visceral fat depots as compared to young *Ldlr* ^*−/−*^ mice (Fig. S2B).

**Figure 4:**
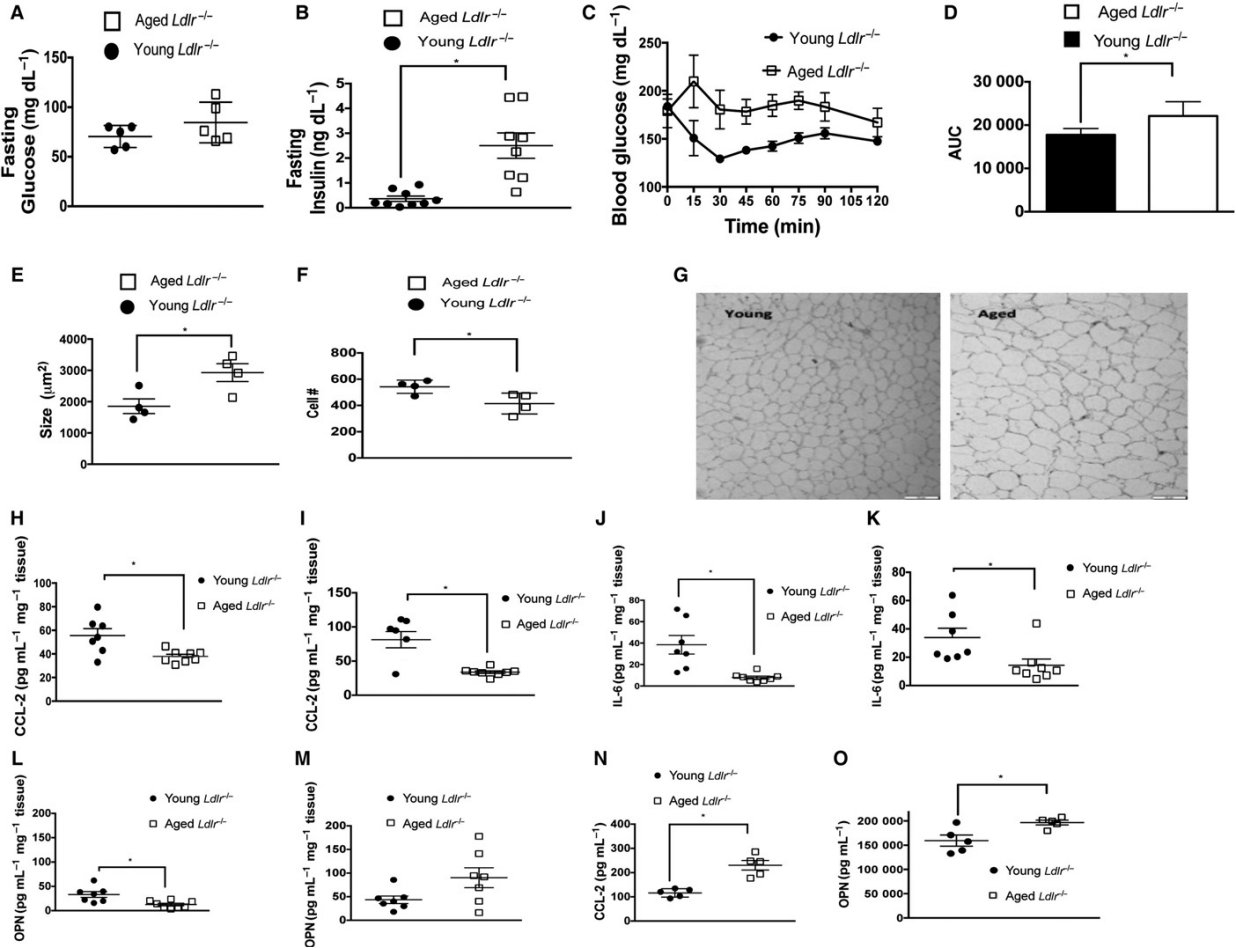
Aging leads to insulin resistance during atherogenesis. (A) Fasting blood glucose was obtained from young (2 months of age) and aged (12 months of age) *Ldlr* ^−/−^ mice administered a HFD for an additional 6 weeks. Glucose was measured via glucometer. *P < 0.01 (*t*‐test). (B) Fasting blood insulin was measured by ELISA obtained from young (2 months of age) and aged (12 months of age), *Ldlr* ^−/−^ mice administered a HFD for an additional 6 weeks. *P < 0.01 (*t*‐test). (C–D) Insulin tolerance test was performed in aged and young *Ldlr* ^−/−^ mice after a 4‐h fast. (D) Area under the curve (AUC) for D, *P* < 0.01 (Mann–Whitney), *n* = 5 mice/group. (E–F) Adipocytes were obtained from the epididymal (visceral) tissue of aged and young *Ldlr* ^−/−^ after 1‐month HFD. Cellularity and size were measured. *P* < 0.01 (*t*‐test). (G) Representative phase contrast micrographs show larger epididymal adipocytes in aged *Ldlr* ^−/−^ mice vs. young mice 1 month after HFD. 200× magnification. (H–M) Adipose tissue was obtained from the subcutaneous (H, J and L) and perigonadal (visceral) fat depots (I, K and M) from aged or young *Ldlr* ^*−/−*^ mice after 1 month of a HFD and cultured *ex vivo*. CCL‐2, IL‐6, and OPN measured in the culture supernatants via ELISA. *P < 0.05. (N–O) Plasma was obtained from young (2 months of age) and aged (12 months of age) *Ldlr* ^−/−^ mice after they were administered a HFD for 1 month. CCL‐2 (N) and OPN (O) were measured in the plasma by ELISA. *P* < 0.01 (*t*‐test).

We next examined whether aged *Ldlr* ^*−/−*^ mice exhibited impaired glucose and insulin levels. We found that fasting blood glucose levels were similar between young and aged *Ldlr* ^*−/−*^ mice after 6 weeks of the HFD (Fig. 4A). However, aged *Ldlr* ^*−/−*^ mice exhibited increased fasting insulin levels (Fig. 4B) and higher glucose levels in response to an insulin tolerance test as compared to young *Ldlr* ^*−/−*^ mice (Fig. 4C–D). Aged WT mice fed a HFD exhibited a larger body weight (Fig. S3A) but similar fasting insulin levels and a similar response in an insulin tolerance test, although the aged mice displayed a slightly slower recovery response during the test (Fig. S3B–D). Adipose tissue from aged *Ldlr* ^*−/−*^ mice fed a HFD for 1 month showed adipocyte hypertrophy reflected in a decrease in cellularity and an increase in cellular size as compared to similarly treated young *Ldlr* ^*−/−*^ mice (Fig. 4E–G), morphological features that associate with insulin resistance.

Given the increased insulin levels and reduced insulin sensitivity in aged *Ldlr* ^*−/−*^ mice, we examined whether adipose tissue from young or aged *Ldlr* ^*−/−*^ mice exhibited an altered ability to produce CCL‐2, IL‐6 and OPN, inflammatory proteins, which have all been implicated in insulin resistance (Kanda *et al*., 2006; Pedersen & Febbraio, 2007; Kiefer *et al*., 2010). Adipose tissue harvested from the visceral and subcutaneous depots of aged *Ldlr* ^*−/−*^ mice secreted lower amounts on a per gram basis of all of these mediators compared to the adipose tissue from young mice 1 month after initiation of a HFD (Fig. 4H–M). An exception to this was OPN secreted by the visceral depot, which showed no difference between young and aged *Ldlr* ^*−/−*^ mice (Fig. 4M). Plasma levels of CCL‐2 levels were increased twofold and OPN levels were increased 10–20% in aged *Ldlr* ^*−/−*^ mice compared to young counterparts after 1 month of a HFD (Fig. 4N–O). However, IL‐6 was not detectable in the plasma, nor was TNF‐α, IL‐17, CCL‐5, and IL‐1β (data not shown). Adiponectin and serum amyloid A (SAA) levels have also been associated with insulin resistance (Scheja *et al*., 2008; Ding *et al*., 2012). However, we did not find any differences in plasma adiponectin or SAA levels between young and aged *Ldlr* ^*−/−*^ mice after 1 month of a HFD (Fig. S3C–D). Overall, these data have revealed that aged *Ldlr* ^*−/−*^ mice exhibit insulin resistance during HFD, which associates with elevated plasma levels of CCL‐2 and OPN.

### Hemodynamic parameter assessment in aged and young *Ldlr^−/−^* mice

Given that insulin resistance is associated with hypertension, we next assessed blood pressure (BP) in the young and aged cohorts prior to and after initiation of the HFD. After inserting ambulatory monitors into mice and letting the mice rest for several days, we recorded ambulatory BP continuously over several days. On the chow diet, aged *Ldlr* ^*−/−*^ mice exhibited a 10–20 mmHg increase in diastolic BP and a 10 mmHg increase in systolic BP (Fig. S4A). This was maintained during the first week of the HFD. However, after 3 weeks on the HFD the BP in young mice increased so that the BPs were similar between the cohorts (Fig. S4A). Despite the initial increase in BP in the aged *Ldlr* ^*−/−*^ mice, noninvasive imaging via echocardiography did not discern any evidence of increased left ventricular (LV) mass or altered systolic LV function in the aged cohort after 3 months on HFD as compared to the young cohort (Fig. S4B–C). Overall, as compared to young mice, aged *Ldlr* ^*−/−*^ mice exhibit increased BP and higher LDL levels on a chow diet, and insulin resistance on a HFD, providing evidence that aging leads to metabolic dysfunction during atherogenesis.

### Stromal and Hematopoietic factors contribute to age‐enhanced atherosclerosis

A prior study indicated that adoptive transfer of aged bone marrow into nonirradiated young *ApoE* ^−/−^ mice was less effective at slowing the progression of atherosclerosis than young bone marrow (Rauscher *et al*., 2003). Hence, we conducted a bone marrow transplant (BMT) study to determine whether age‐enhanced atherosclerosis is due to stromal or hematopoietic factors. In agreement with prior work (Geiger *et al*., 2013), we found a slight increase in the proportion of hematopoietic stem cells in the bone marrow with aging (Fig. S5A). We then determined that bone marrow cells from aged WT CD45.2^+^ mice were similarly able to engraft into young irradiated CD45.1^+^ WT mice as young WT CD45.2^+^ bone marrow cells (Fig. S5B).

We next determined whether aged or young *Ldlr* ^*−/−*^ lethally irradiated mice exhibited a similar ability to be engrafted by bone marrow from young WT, CD45.1^+^ donors. We found that 2 months after bone marrow transplantation with young CD45.1^+^ bone marrow cells and a chow‐fed diet, aged *Ldlr* ^*−/−*^ mice exhibited a similar degree of engraftment in the spleen (91%) as young *Ldlr* ^*−/−*^ mice (90%) (Fig. S5C). In the aorta of engrafted *Ldlr* ^*−/−*^ mice, the majority of immune cells were of donor origin (i.e. CD45.1^+^ cells, young *Ldlr* ^*−/−*^ mice: 94%; aged *Ldlr* ^*−/−*^ mice: 92%) although there was a reduction in the proportion of donor CD45.1^+^ cells in the aortas of aged *Ldlr* ^*−/−*^ mice compared to young *Ldlr* ^*−/−*^ mice (63% vs. 76%), which was mainly due to an increase in nonimmune cells (i.e. CD45^−ve^ cells) in the aorta of aged mice compared to young mice (Fig. S5C). These nonimmune cells may be VSMC, which are known to exhibit increased proliferative capacity with aging (Wang *et al*., 2012). These data show that aged *Ldlr* ^*−/−*^ mice were similarly engrafted after BMT as young *Ldlr* ^*−/−*^ mice.

**Figure 5:**
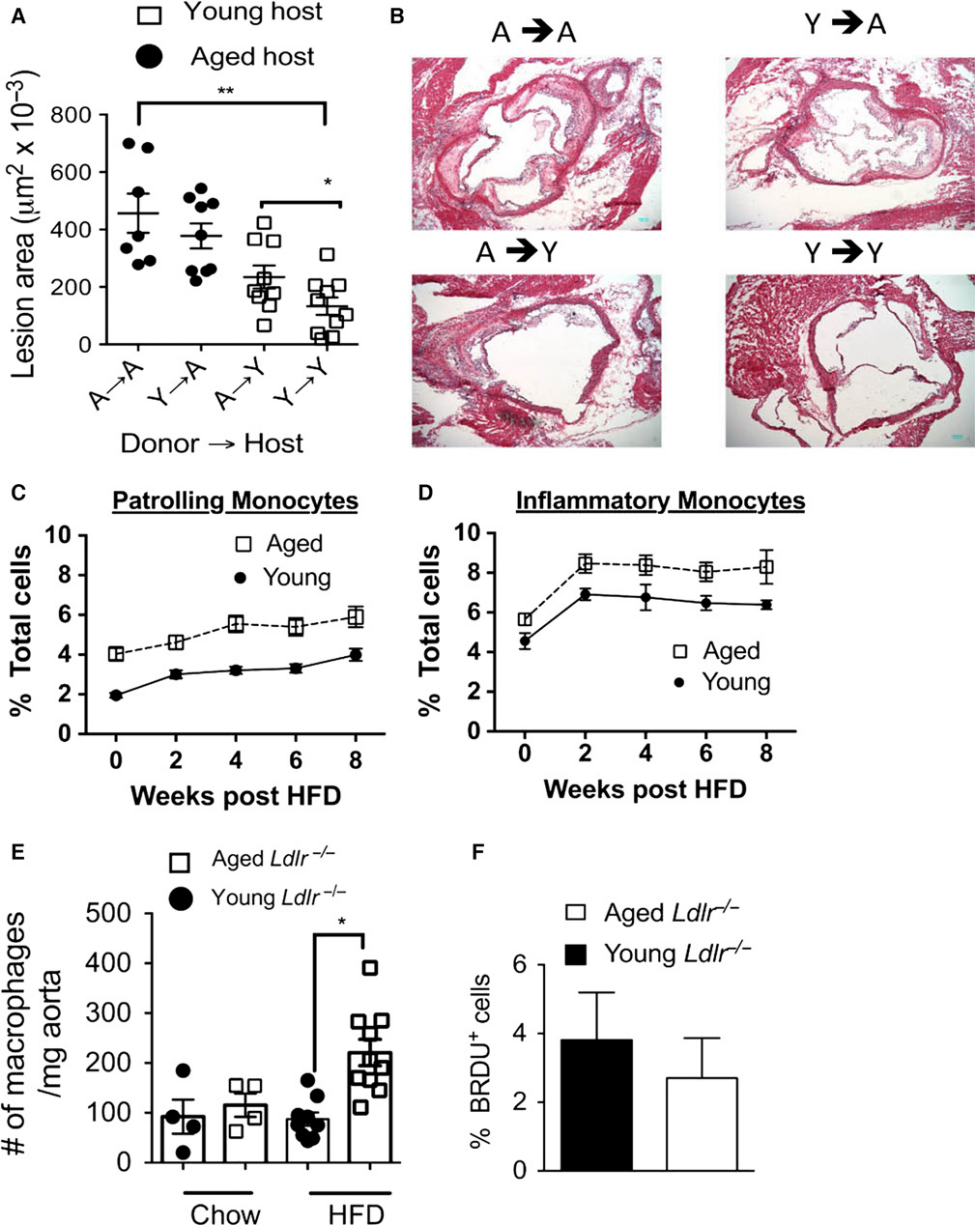
Aging induces a peripheral monocytosis and increased macrophage numbers within the aorta. (A–B) Young (2 months of age) and aged (12 months of age) male *Ldlr* ^−/−^ mice were lethally irradiated and then reconstituted i.v. with 1 × 10^7^ bone marrow cells from age‐matched or age‐mismatched *Ldlr* ^*−/−*^ donor mice. The mice were rested for 2 months and then fed a HFD for 2 months. Atherosclerotic lesion assessment was determined by lesion size in the aortic root by H&E. Blue scale bar = 100 μm. *P < 0.05; **P < 0.001 (*t*‐test). (C–D) Young (2 months) and aged (12 months) male *Ldlr* ^−/−^ mice were placed on a HFD diet for the indicated time. At weekly intervals, peripheral blood was obtained and peripheral blood mononuclear cells were isolated. The cells were stained with fluorescently labeled monoclonal antibodies to assess patrolling monocytes CD115^+^, Ly6c^lo^ cells) (C) and inflammatory monocytes (CD115^+^, Ly6c^hi^ cells) (D). *N* = 15–20 mice group, error bars = SEM. Each data point per time interval was significantly different between the young and aged groups (*P* < 0.001, *t*‐test). (E) The aortas of *Ldlr* ^−/−^ young and aged mice maintained on a chow diet or given a HFD for 1 month were harvested, digested and their cellular suspensions were obtained. The cells were stained with fluorescently tagged monoclonal antibodies and fluorescent data acquired via flow cytometry. Macrophages defined as CD11b^+^, F4/80^+^ GR1^−^. *P < 0.01 (*t*‐test). (F) As per E but mice were administered BrdU in the drinking water up to 1 week prior to tissue harvest.

We next lethally irradiated aged and young *Ldlr* ^*−/−*^ mice and infused them i.v., with 1 × 10^7^ bone marrow cells from either age‐matched or age‐mismatched *Ldlr* ^*−/−*^ bone marrow donors. Mice were left to rest for 2 months after bone marrow transplantation and then were fed a HFD for another 2 months after which time fasting plasma were obtained and cholesterol levels measured. Total cholesterol levels were similar among the chimeric groups (Fig. S5D). The mice were then euthanized and the degree of atherosclerosis was measured in the aortic root. We found that aged *Ldlr* ^*−/−*^ mice that received aged *Ldlr* ^*−/−*^ bone marrow exhibited larger atherosclerotic lesions than young mice that received young bone marrow (Fig. 5A–B), recapitulating the phenotype of nontransplanted aged and young mice (Fig. 1). Young mice that received aged bone marrow cells exhibited a significant 20–30% increase in lesion size compared to young mice that received young bone marrow cells, although this increase was still significantly smaller than that for aged mice that were infused with aged bone marrow (Fig. 5A). Aged mice that received young bone marrow exhibited a similar atherosclerotic lesion size as the aged‐to‐aged chimera (Fig. 5A). Thus, these data demonstrate that aged bone marrow cells contribute, but are not sufficient, to age‐enhanced atherosclerosis. Furthermore, the data also reveal that the presence of young bone marrow does not modulate the progression of atherosclerosis in aged hosts.

### Aged *Ldlr^−/−^* mice exhibit enhanced monocytosis and increased macrophage numbers within the aortas during HFD

A HFD diet is known to lead to a peripheral monocytosis in atherosclerotic prone mice (Swirski *et al*., 2007; Tacke *et al*., 2007). Given this, we next enumerated the number of circulating monocytes (defined as CD115^+^ cells) in young and aged *Ldlr* ^*−/−*^ mice before and during the HFD via antibody staining and subsequent flow cytometric analysis. We found that before initiation of the HFD, aged *Ldlr* ^*−/−*^ mice exhibited increased proportions of both patrolling and inflammatory monocytes in the peripheral blood as compared to young *Ldlr* ^*−/−*^ mice (Fig. 5C–D). These changes persisted during the HFD, with aged mice maintaining an enhanced proportion of inflammatory monocytes as compared to young mice (Fig. 5C). Similar findings were found in aged female *Ldlr* ^*−/−*^ mice fed a HFD as compared to similarly treated young female *Ldlr* ^*−/−*^ mice (Fig. S6A–B). Additionally, WT aged mice exhibited increased proportions of both subpopulations of monocytes during HFD as compared to young WT mice fed a HFD (Fig. S6C–D). Thus, these data reveal that aging enhances an inflammatory monocytosis during a HFD.

**Figure 6:**
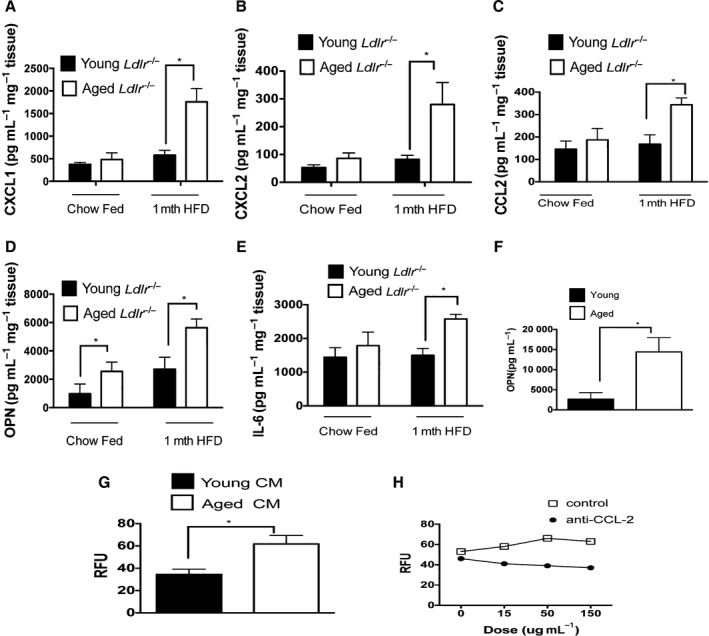
Aging induces increased aortic production of inflammatory molecules and induces increased monocyte chemotaxis. (A–E) The aortas from young (3 months of age) and aged (13 months) *Ldlr* ^−/−^ male mice on chow diet or after 1 month of a HFD were cultured for 12 h and the indicated chemokine or cytokine were measured in the culture supernatant via ELISA. A: CXCL1, B: CXCL2: C: CCL‐2, D: OPN, E: IL‐6. *N* = 5–10 mice/group. (F) VSMC were propagated from the aortas of aged (15 months) and young (2 months) C57BL/6 WT mice. After overnight culture, OPN was measured in the culture supernatant via ELISA. *P < 0.01 (*t*‐test). Representative experiment that was repeated twice with similar results, *n* = 2–3 biological replicates/experimental group. (G) Aortas from young (2 months of age) or aged (12 month of age) *Ldlr* ^−/−^ male mice given a HFD for an additional month were cultured *ex vivo* for 12 h and the conditioned media were obtained and placed in the Boyden chamber. Monocytes isolated from young WT C57BL/6 mice were seeded into the chamber and their ability to chemotax toward the aged or young conditioned media was assessed. RFU = relative fluorescent units. *N* = 5 biological replicates/group, *P < 0.01 (*t*‐test). (H) Aortas from aged *Ldlr* ^−/−^ mice that were fed a HFD for 1 month were cultured *ex vivo* for 12 h and the culture media were used in the Boyden chamber seeded with monocytes isolated from the bone marrow from young WT mice. Increasing doses of an anti‐CCL‐2 antibody or isotype control were added to the conditioned media and monocyte chemotaxis was assessed. Representative of one experiment that was repeated once with similar results.

We next obtained aortas from young and aged *Ldlr* ^*−/−*^ mice before and during the HFD period, enzymatically digested them, and enumerated the numbers of macrophages (defined as F4/80^+^CD11b^+^ cells) via antibody staining and subsequent flow cytometric analysis as previously reported (Galkina *et al*., 2006). Prior to HFD, aged and young *Ldlr* ^*−/−*^ mice exhibited similar concentrations of macrophages within the aortas (Fig. 5E). One month after the HFD, we observed increased concentrations of macrophages in aged aortas, with aged aortas exhibiting a significantly threefold higher macrophage numbers than young aortas (Fig. 5E). However, we found similar levels of macrophage proliferation within the aortas of aged and young mice fed a HFD (Fig. 5F), indicating that increased macrophage accumulation in the age aortas was not likely due to increased cell turnover.

### Microarray analysis of aortas of young and aged *Ldlr^−/−^* reveals multiple up and down gene sets

We next examined the global changes in gene expression within the aorta with aging, prior to atherogenesis, and then after atherosclerosis development. For this purpose, we obtained mRNA from the aortas of the following aged (15 months of age) and young mice (5 months of age): i) WT mice on a chow diet; ii) *Ldlr* ^*−/−*^ mice on a chow diet; and iii) aged *Ldlr* ^*−/−*^ mice administered a HFD for 3 months. All the differentially regulated genes between young and aged mice in each of these three groups are shown in Table S1. The differentially displayed genes between the young and aged aortas in each group are displayed in a heat map and indicate that there were larger differences between expressed genes in the aged vs. young *Ldlr* ^*−/−*^ mice maintained on a chow diet compared to the other groups (Fig. S7). Thus, we focused on this group for our gene set analysis using QuSAGE gene set enrichment analysis.

We observed that gene sets that were upregulated within the aorta with aging included viral invasion and host response, platelet adhesion; and extracellular matrix degradation (Table S2). Downregulated gene sets within aging aorta included viral resistance (apolipoprotein B mRNA editing enzyme catalytic polypeptide 3 (family); DNA replication; autophagy; and cell surface anchor proteins (glycosylphosphatidylinositol) (Table S3). Overall, these gene expression data reveal that aging has multiple complex effects within the vasculature during atherogenesis.

### Aortas of aged *Ldlr^−/−^* mice secrete higher levels of chemokines and the proinflammatory cytokine IL‐6

Given the increase in aortic macrophages and the complex alteration in gene sets within the aorta in aged atherosclerotic aortas, we next determined whether aged atherosclerotic aortas secreted more chemokines and inflammatory cytokines as compared to young aortas. For this purpose, we obtained aortas from young (3 months of age) and aged (13 months of age) *Ldlr* ^*−/−*^ mice either maintained on a chow diet or given a HFD for the last month. After we flushed the aortas and removed any periaortic fat and connective tissue, we cultured the aortas *ex vivo* for 12 h to measure the production of chemokines and inflammatory cytokines. Conditioned media from aged aortas exhibited a higher concentration (two‐ to tenfold higher) of CCL‐2, CXCL1 and CXCL2 (both neutrophil chemokines), and OPN (a monocyte chemokine), than aortas harvested from young mice (Fig. 6A–D). We also found that aortas from aged *Ldlr* ^*−/−*^ mice secreted a 40% increase in IL‐6, a proinflammatory cytokine that exacerbates atherosclerosis (Fig. 6E) (Huber *et al*., 1999). Aortas from chow‐fed mice generally produced lower concentrations of the above inflammatory mediators although aged aortas secreted twice as much OPN, than aortas from young mice that were fed a chow‐fed diet (Fig. 6D).

To identify potential vascular cellular sources that contribute to age‐elevated production of inflammatory proteins by the aorta, we propagated VSMC from the aortas of either young (i.e. 2 months of age) or aged (15 months of age) WT mice as described in our prior work (Song *et al*., 2012). We found that aged VSMC produced 2–3 higher levels of OPN under basal conditions than young VSMC (Fig. 6F), in agreement with our prior study that found similar age‐dependent elevations in the production of IL‐6 and CCL‐2 by VSMC from WT mice (Song *et al*., 2012). These data indicate that prior to the development of atherosclerosis, aging VSMC elicit the production of inflammatory proteins.

### Monocytes from aged *Ldlr^−/−^* mice do not exhibit intrinsic chemotaxis alterations

As an enhanced cell‐intrinsic ability of monocytes to chemotax with aging could explain the increased peripheral monocytes and subsequent increased numbers of macrophages into the aorta after 1 month of the HFD with aging, we isolated young and aged monocytes from *Ldlr* ^*−/−*^ mice and examined their ability to chemotax in response to CCL‐2 in an *in vitro* chemotaxis assay. We found that monocytes isolated from the bone marrow of aged *Ldlr* ^*−/−*^ mice exhibited a similar ability to chemotax as young monocytes (Fig. S8A). We then harvested the aortas from young *Ldlr* ^*−/−*^ mice administered a HFD for 1 month. We cultured the tissue *ex vivo* for 12 h as the conditioned media from atherosclerotic aortas contains a cocktail of inflammatory chemokines (Fig. 6A–E). The conditioned media induced potent monocyte chemotaxis. However, monocytes harvested from aged or young *Ldlr* ^*−/−*^ mice were equally able to chemotaxis toward the aortic conditioned media (Fig. S8B).

We also assessed the gene expression of several inflammatory proteins, specifically IL‐1β, TNF‐α, OPN, and IL‐6, and an innate immune signal adaptor, MyD88, in aged and young bone marrow monocytes isolated from *Ldlr* ^*−/−*^ mice maintained on a chow diet. Except for IL‐1β, all of the genes were expressed at low levels relative to the house keeping gene, β‐actin (Fig. S8C). Monocytes isolated from aged mice exhibited a fivefold lower expression IL‐1β than monocytes from young mice (Fig. S8C).

On a HFD, we assessed the surface expression of CCR2 and CCR5. CCR2 is a chemokine that is the receptor for CCL‐2 and is involved in transmigration into tissues (Moore *et al*., 2013). CCR5 is a chemokine receptor involved in monocyte arrest on the vasculature. We found that CCR2 was highly expressed in inflammatory but not in patrolling monocytes (Fig. S8D). Within each population of monocytes there was no evidence of altered expression of CCR2 due to age (Fig. S9D). Similarly, CCR5 was expressed at low levels in both patrolling and inflammatory monocytes with no differences between the age groups (Fig. S8E). Similar results were noted in female *Ldlr* ^*−/−*^ mice (Fig. S8D–E). Overall, these data reveal that aging neither enhances the cell‐intrinsic ability of monocytes to chemotax, nor upregulates the inflammatory profile or chemokine receptors on monocytes.

### Conditioned media from aortas from aged *Ldlr^−/−^* mice exhibit enhanced ability to induce monocyte chemotaxis

As aged aortas produce higher levels of chemokines than young aortas, we assessed whether the conditioned media produced by aged aortas induced a higher degree of monocyte chemotaxis than conditioned media produced by young aortas. Bone marrow monocytes were purified from young WT mice and were seeded in the chemotaxis assay with conditioned media from either young or aged aortas from *Ldlr* ^*−/−*^mice that were fed a HFD for 1 month. We found that conditioned media from aged *Ldlr* ^*−/−*^ aortas induced a higher degree of monocyte chemotaxis than the conditioned media from young aortas (Fig. 6G). When we applied an anti‐CCL‐2 inhibitory antibody to the conditioned media from the aged atherosclerotic aortas, we noted that this diminished the ability of the conditioned media to induce monocyte chemotaxis by 30% (Fig. 6H), indicating that the age‐elevated levels of CCL‐2 contributed to monocyte chemotaxis toward the atherosclerotic aorta. Overall, our study has revealed that the inflammatory proteins secreted by the aged atherosclerotic aorta act as extrinsic factors to enhance monocyte chemotaxis.

## Discussion

Using an established model of atherosclerosis, the *Ldlr* ^*−/−*^ mouse, we have uncovered that aging has complex effects on atherogenesis. Consistent with prior studies using aging *Apoe* ^*−/−*^ mice (Pereira *et al*., 2010; Vendrov *et al*., 2015), our study shows that aging associates with increased atherosclerotic lesion size. Aging atherosclerotic mice exhibit features consistent with metabolic syndrome: elevated BP increased lipid levels (on a chow diet), insulin resistance after a HFD, fatty liver, and increased body weight. These metabolic alterations with aging may impact atherogenesis via increased lipid deposition within the vasculature and changes in hemodynamics. Thus, our study has revealed that metabolic dysfunction is one of the factors by which aging could enhance atherosclerosis.

We also discovered that aging induces an increased peripheral monocytosis, including inflammatory monocytes, during atherogenesis. Human monocytes exhibit dysregulated inflammatory responses with aging consisting of both elevated (i.e. increased TNF‐α) and impaired (i.e. decreased IL‐1β) responses (Sadeghi *et al*., 1999; Hearps *et al*., 2012), both of which could contribute to atherosclerosis. Yet we did not discern any monocyte intrinsic enhancements, either in chemotaxis, basal inflammatory responses, or chemokine receptor expression during HFD that could explain either the increased monocytosis, the increased accumulation of macrophages within the aorta, or the enhanced inflammatory response of the atherosclerotic aorta with aging.

To uncover monocyte‐extrinsic factors that could lead to enhanced macrophage accumulation within the aging aorta, we assessed the production of monocyte chemokines by the aorta and found that aged atherosclerotic aortas produced higher concentrations of monocyte chemokines (e.g. CCL‐2 and OPN) and IL‐6 than young aortas. Importantly, aging atherosclerotic aortas induced a higher degree of monocyte chemotaxis than young aortas. CCL‐2 contributed to this age‐increased monocyte chemoattraction, a chemokine that has been shown to be critical for atherogenesis in young mice (Gu *et al*., 1998). However, CCL‐2 was not sufficient for age‐enhanced monocyte chemotaxis, indicating that multiple inflammatory factors produced by the aging aorta contribute to monocyte recruitment and atherogenesis with aging. Importantly, there are multiple steps involved in monocyte recruitment into the aorta including monocyte arrest, adhesion to the vascular wall and transmigration into the tissue (Moore *et al*., 2013; Hilgendorf *et al*., 2015). It is possible that the aged vasculature could promote macrophage accumulation through any of these complex steps, which will require future investigation. Inflammatory monocytes that enter the arterial wall develop into M1 macrophages, which are enriched within atherosclerotic lesions and produce proinflammatory cytokines (Moore *et al*., 2013). M2 macrophages are associated with atherosclerotic lesion regression and the production of anti‐inflammatory cytokines such as IL‐10. Future studies will be required to determine how aging impacts macrophage biology within the aorta during atherogenesis and atherosclerosis regression. Overall, our study has revealed that the enhanced inflammatory environment within the aging atherosclerotic aorta acts as a monocyte‐extrinsic factor that promotes monocyte chemotaxis during atherogenesis.

To identify a potential vascular source of inflammatory proteins during age‐enhanced atherosclerosis, we propagated VSMC from the aortas of disease‐free, WT mice. VSMC from aged mice exhibited higher OPN production than young VSMC under basal conditions. This observation is consistent with our prior work that found that in disease‐free WT aged mice, aging leads to a two‐ to threefold increase in the basal production of IL‐6 and CCL‐2 by VSMC (Song *et al*., 2012), a phenotype consistent with a senescent secretory phenotype (Tchkonia *et al*., 2013). Our findings are also consistent with prior studies in disease‐free rodents, nonhuman primates, and humans. These studies found that CCL‐2, IL‐6, and OPN are increased by the aging vasculature (Spinetti *et al*., 2004; Marchand *et al*., 2011; Csiszar *et al*., 2012). Our current study had determined that these enhanced inflammatory features of the aging vasculature persist during atherogenesis. We previously also observed that the age‐dependent increase in the production of IL‐6 by VSMC was dependent on the innate immune adaptor protein, MyD88 (Song *et al*., 2012). Future studies will be required employing mice in which key inflammatory signal transducers, such as MyD88, are conditionally deleted within vascular cells to determine the role such pathways play in age‐enhanced atherosclerosis.

OPN, IL‐6 and CCL‐2 have all been implicated in insulin resistance (Kanda *et al*., 2006; Pedersen & Febbraio, 2007; Kiefer *et al*., 2010). Thus, the enhanced aortic production of these inflammatory proteins by the aging atherosclerotic aorta may contribute to the insulin resistance and metabolic syndrome found in our study. It is also possible that other sources of inflammatory proteins, such as the adipose tissue, contribute to insulin resistance with aging during atherosclerosis. This notion is supported by prior work in WT aged mice that demonstrates that macrophages from aged adipose tissue exhibit enhanced inflammatory responses and that a HFD in young mice induces an inflammatory phenotype in macrophages found within adipose tissue (Lumeng *et al*., 2007, 2011).

Our current study reveals that aging *Ldlr* ^*−/−*^ mice exhibit twofold higher LDL plasma levels during a chow diet than young *Ldlr* ^*−/−*^ mice. These increased LDL levels over time could contribute to atherosclerosis. We cannot distinguish the effects of these elevated LDL levels and intrinsic effects of aging within the vasculature or immune system on atherosclerosis. Future studies will be required in which aging WT mice are rendered hyperlipidemic (e.g. by downregulating the LDL receptor) and then administered a HFD to determine how different factors, including the vasculature, may predispose to atherosclerosis with aging. Humans age with increased lipid levels and hypertension during atherogenesis. Thus, the elevated BP and LDL levels that we have noted with aging in our study may, in part, mimic atherogenesis in aging humans.

Our microarray data have discovered that genes in the autophagy pathway are decreased with aging within the vasculature. Aging has been shown to impair autophagy in a wide variety of tissue although how aging impacts autophagy within the vasculature is unclear (Dutta *et al*., 2012). It is possible that impaired autophagy compromises effective removal of damaged organelles within the vasculature and this could contribute to the age‐enhanced inflammatory responses of VSMC with aging. Prior work in aging *ApoE* ^−/−^ mice indicates that autophagy, manifest as elevated P62 levels, is reduced in the aging aortas (Razani *et al*., 2012). Furthermore, intact autophagy pathways in macrophages contribute to reducing atherosclerosis disease burden (Liao *et al*., 2012; Razani *et al*., 2012). Future studies will be required to elucidate the autophagic pathways impacted by aging within the vasculature, the cell types involved, and the contribution to age‐enhanced atherosclerosis.

Our study found that the key effects of aging on atherogenesis, notably increased atherosclerotic lesion size and monocytosis, are evident in both genders. A prior study in *Apoe* ^*−/−*^ mice found that young (<6 months of age) female mice fed a high‐fat diet exhibit smaller atherosclerotic lesion size than male mice (Chiba, 2011). Yet another study reported that this phenotype was maintained in aged (18 months of age) *Apoe* ^*−/−*^ mice administered a chow diet (Pereira *et al*., 2010). In contrast, our results indicate that young and middle aged female *Ldlr* ^*−/−*^ mice have larger lesions than age‐matched male mice (Fig. 1). Future studies will be needed to determine the effect of gender on aging in detail in the different models of atherosclerosis.

In conclusion, our study has revealed that the aging vasculature acts as a major source of inflammatory molecules that enhance monocyte chemotaxis and macrophage accumulation within the aorta during atherosclerosis. Thus, our work has uncovered novel insights into how the complex effects of aging impact atherosclerosis.

## Experimental procedures

### Mice and diet

C57BL/6 *Ldlr* ^−/−^ mice (stock # 002207) were purchased from Jackson Laboratories (Bar Harbor, ME, USA) bred and aged in the same room of our animal facility. Mice were maintained under pathogen‐free conditions and administered a chow diet or a HFD (42% fat) from Harlan (cat# TD.88137) for designated time points. Mice were not employed in the study if they exhibited signs of illness (i.e. evidence of skin lesions, reduced mobility, grooming, feeding, and weight loss). Mice were weighed monthly during the HFD period. WT aged and young C57BL/6 mice were obtained from the NIA rodent facility. C57BL/6 CD45.1^+^ mice were obtained from The Jackson Laboratory (stock #002014).

### Atherosclerosis disease measurement

Ascending aortas were harvested and sectioned at the aortic root and were assessed for lesion size by hematoxylin and eosin (H&E) staining. Total lesion size and size of acellular lesion area, a correlative of necrotic core size (Fernandez‐Hernando *et al*., 2007), was measured in sections stained with H& E using imagej software, NIH, USA. Photographs of the ascending aortas of mice were taken with a UCMOS series microscope camera. Lesion calcification was obtained after staining via alizarin red (Electron Microscopy Sciences) as previously reported (Sun *et al*., 2012).

### Fasting lipid, glucose and insulin levels

Plasma lipid levels were obtained from mice after an overnight fast, and lipid levels were assessed by HPLC. Fasting cholesterol levels were measured by ELISA (Wako Pure Chemicals, Tokyo, Japan). Fasting plasma glucose levels were measured by a glucometer (Bayer). Fasting insulin was measured by ELISA (Crystal Chem Inc., Downers Grove, IL, USA). The insulin tolerance test was assessed by administering insulin at 0.75 μ kg^−1^ body weight i.p. and determining blood glucose levels at indicated time points after injection.

### Aortic culture, ELISA, monocyte cell purification, and chemotaxis assay

Prior to dissecting the descending aortas, mouse hearts were perfused with cold PBS. The aortas were harvested after removing the periaortic fat tissue and connective tissue under a Zeiss dissecting microscope, Carl Zeiss Microscopy, LLC, United States. The aortas were then cultured for 12 h *ex vivo* in M199 (20% fetal bovine serum) media. Analytes within the supernatants or plasma were measured by ELISA for OPN, CCL‐2, and CXCL1/2 (R&D Systems, Minneapolis, MN, USA). To examine whether the supernatants produced by the *ex vivo* aortic culture from *Ldlr* ^−/−^ mice induced a monocyte chemotaxis, we collected conditioned media from the cultured aortas from young and aged *Ldlr* ^−/−^ mice and added the media to a Boyden chamber (Cell Biolabs, San Diego, CA, USA). Bone marrow monocytes purified from C57BL/6 mice (negative selection protocol from StemCell) were seeded into an inset of the chamber that was separated from the conditioned media (or control unconditioned media) by a porous 5 μm membrane. After 3 h, the inset and media were removed and monocytes were detached from the membrane and released into the chamber media. After fluorescently labeling monocytes with LeukoTracker dye, the cells were lysed. Fluorescence in the chamber media was measured via a fluorescent plate reader (Biotek, Winooski, VT, USA), as a readout of monocyte chemotaxis from the inset toward the conditioned media. Anti‐CCL‐2 or isotype control monoclonal antibodies (BD Biosciences, San Diego, CA, USA) were added to the Boyden chamber at the indicated doses.

### Adipose tissue culture and morphometry

See Data S1.

### VSMC culture

VSMC were enzymatically isolated as previously described (Song *et al*., 2012). Briefly, freshly harvested thoracic aortas were washed in PBS and DMEM containing 0.25 μg mL^−1^ amphotericin. After digestion in 1 mg mL^−1^ collagenase II solution at 37 °C for 10 min, adventitia was removed with the aid of a dissecting microscope. The remaining aortas were cut into small pieces and further digested with 2 mg mL^−1^ collagenase II and 0.5 mg mL^−1^ elastase solution for 1 h at 37 °C, with gentle shaking every 10 min. The isolated cells were then washed and plated in complete medium (20% fetal bovine serum, DMEM‐low glucose containing 100 U mL^−1^ penicillin, 100 μg mL^−1^ streptomycin, and 0.25 μg mL^−1^ amphotericin which contained undetectable levels of LPS as measured by Limulus assay) at 37 °C for 14 days.

### BrdU administration, flow cytometry, and tissue digestion

Single‐cell suspensions were obtained from lymphoid tissue (bone marrow or spleens) or aortic tissue after digestion with collagenase per prior work (Galkina *et al*., 2006). The cells were then stained with the following fluorescently tagged monoclonal antibodies where indicated: CD11b, F480, CD115, CD4, CD8, CD19, c‐kit, SCA‐1, NKp46, Ly6c, CD11c, CD11b, F4/80, CCR2, and CCR5. To assess macrophage proliferation, mice were administered drinking water containing bromodeoxyuridine (BrdU) (Sigma, St Louis, Mo, USA) at concentration of 0.8 mg mL^−1^ for 1 week. Aorta single‐cell suspensions were prepared as described above and incorporation of BrdU was measured using APC‐conjugated anti‐BrdU antibodies (eBioscience, San Diego, CA, USA) according to the manufacturer's instructions. Flow cytometric data were acquired by on LSRII flow cytometer and analyzed with flowjo software ,Flowjo, Ashland Or, USA.

### Liver Oil Red O staining

For histological analysis, mouse livers were perfused with PBS and fixed overnight with 4% paraformaldehyde (PFA) at 4 °C. After incubation, livers were washed with 1× PBS, incubated in 30% sucrose for 24 h, embedded in OCT and frozen. Serial sections were cut at 8 μm thickness using a cryostat and stained with red oil for visualization of neutral lipids as previously described (Goedeke *et al*., 2014).

### Bone marrow transplantation

See Data S1.

### Statistical analysis

Means between indicated experimental groups were statistically analyzed by parametric tests (2‐way Student's *t*‐test) or nonparametric tests (Mann–Whitney) using graphpad prism software, GraphPad Software, Inc., La Jolla, CA, USA. A *P*‐value <0.05 was considered significant. Errors shown in figures represent standard error of mean (SEM).

### Regulatory approval

The use of mice in this study was approved by the Yale University IACUC.

## Funding

This study was supported by NIH grant R01AG028082 to DRG.

## Conflict of interest

None.

## Author contributions

DRG conceptualized the study; WD, YS, and DRG systematically analyzed the methods; WD, DRG, ADD, CFH, NR, CW, and NP performed investigation; HM, SK, WD, and DRG performed formal analysis; DRG wrote the original draft; WD, ADD, and CFH reviewed and edited the manuscript; DRG provided resources; and DRG acquired funding.

## Supporting information


**Fig. S1** Assessment of atherosclerosis in advanced aged *Ldlr^−/−^* mice and young and aged WT mice

**Fig. S2** Weight assessment in *Ldlr ^−/−^* mice

**Fig. S3** Assessment of insulin resistance in WT mice fed HFD

**Fig. S4** Hemodynamic parameters

**Fig. S5** Bone marrow transplant parameters

**Fig. S6** Inflammatory monocytosis in young and aged female *Ldlr ^−/−^* mice, and in young and aged WT mice

**Fig. S7** Heatmap of differentially regulated gene between aortas of young and aged mice.

**Fig. S8** Impact of aging on monocyte chemotaxis and basal inflammatory responses.

**Data S1.** Material and Methods.
**Table S1** Lists of genes that were significantly down or upregulated in WT young vs. aged mice on a chow diet, young vs. aged *Ldlr* ^*−/−*^ mice on a chow diet; and young vs. aged *Ldlr* ^*−/−*^ mice fed a HFD for 3 months.
**Table S2** Upregulated gene sets between the aortas of aged and young *Ldlr* ^*−/−*^ in mice maintained on a chow fed diet.
**Table S3** Downregulated gene sets between the aortas of aged and young *Ldlr* ^*−/−*^ in mice maintained on a chow‐fed diet.
