# Supplementary figures and images for: Age‐associated vascular inflammation promotes monocytosis during atherogenesis
Source: Aging Cell. 2016 May 2;15(4):766–77. doi: 10.1111/acel.12488 (PMC4933655; doi:10.1111/acel.12488)

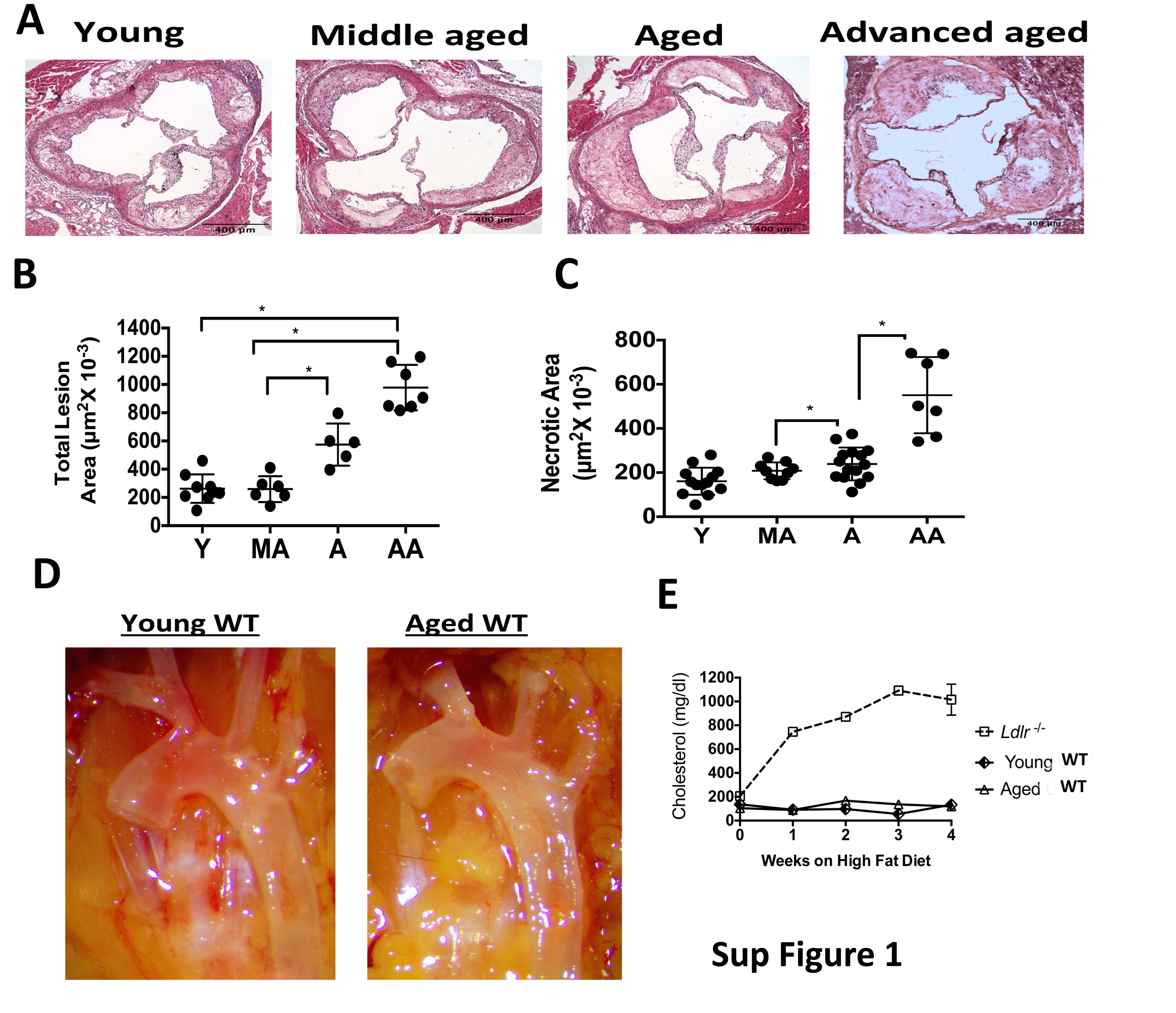

Supplement: Supplementary file 1 — Fig. S1 Assessment of atherosclerosis in advanced aged Ldlr−/− mice and young and aged WT mice [file ACEL-15-766-s001.jpg]

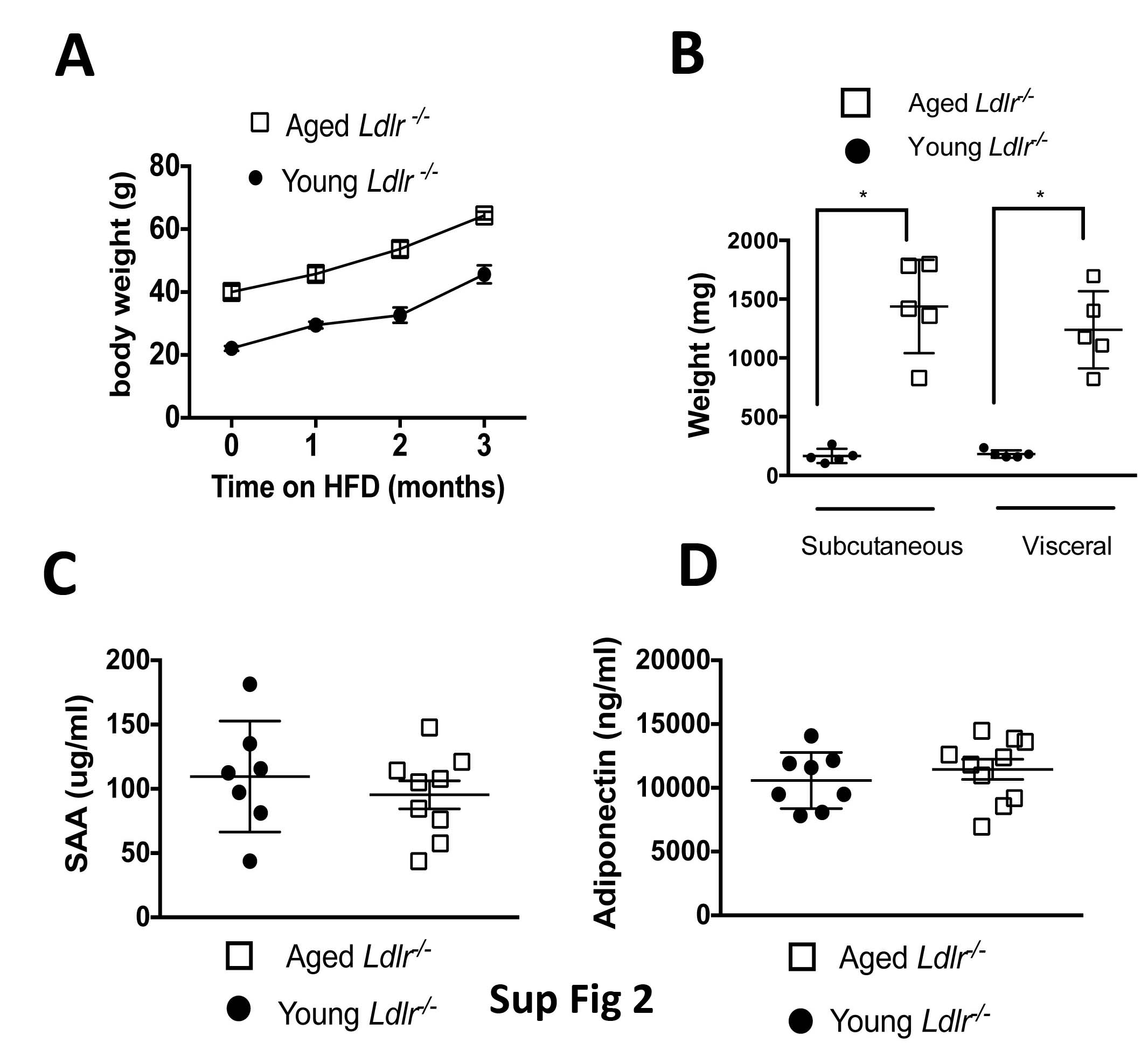

Supplement: Supplementary file 2 — Fig. S2 Weight assessment in Ldlr −/− mice [file ACEL-15-766-s002.jpg]

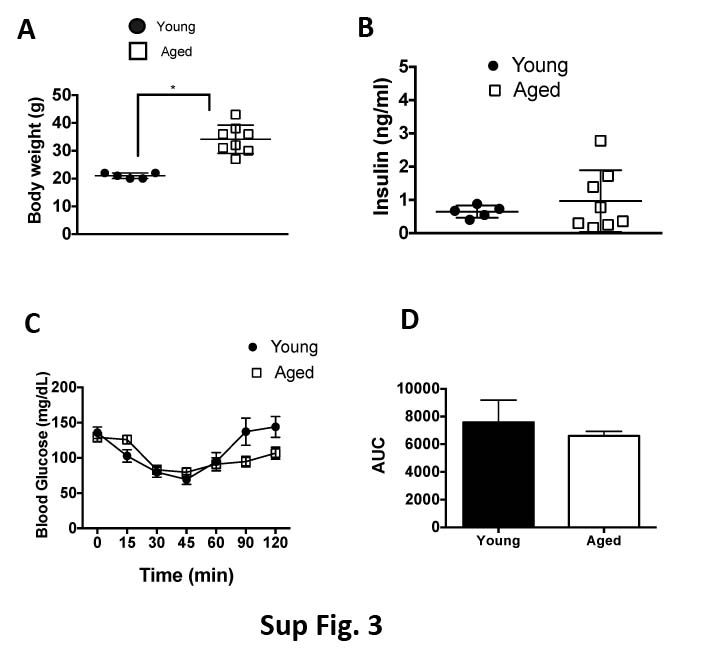

Supplement: Supplementary file 3 — Fig. S3 Assessment of insulin resistance in WT mice fed HFD [file ACEL-15-766-s003.jpg]

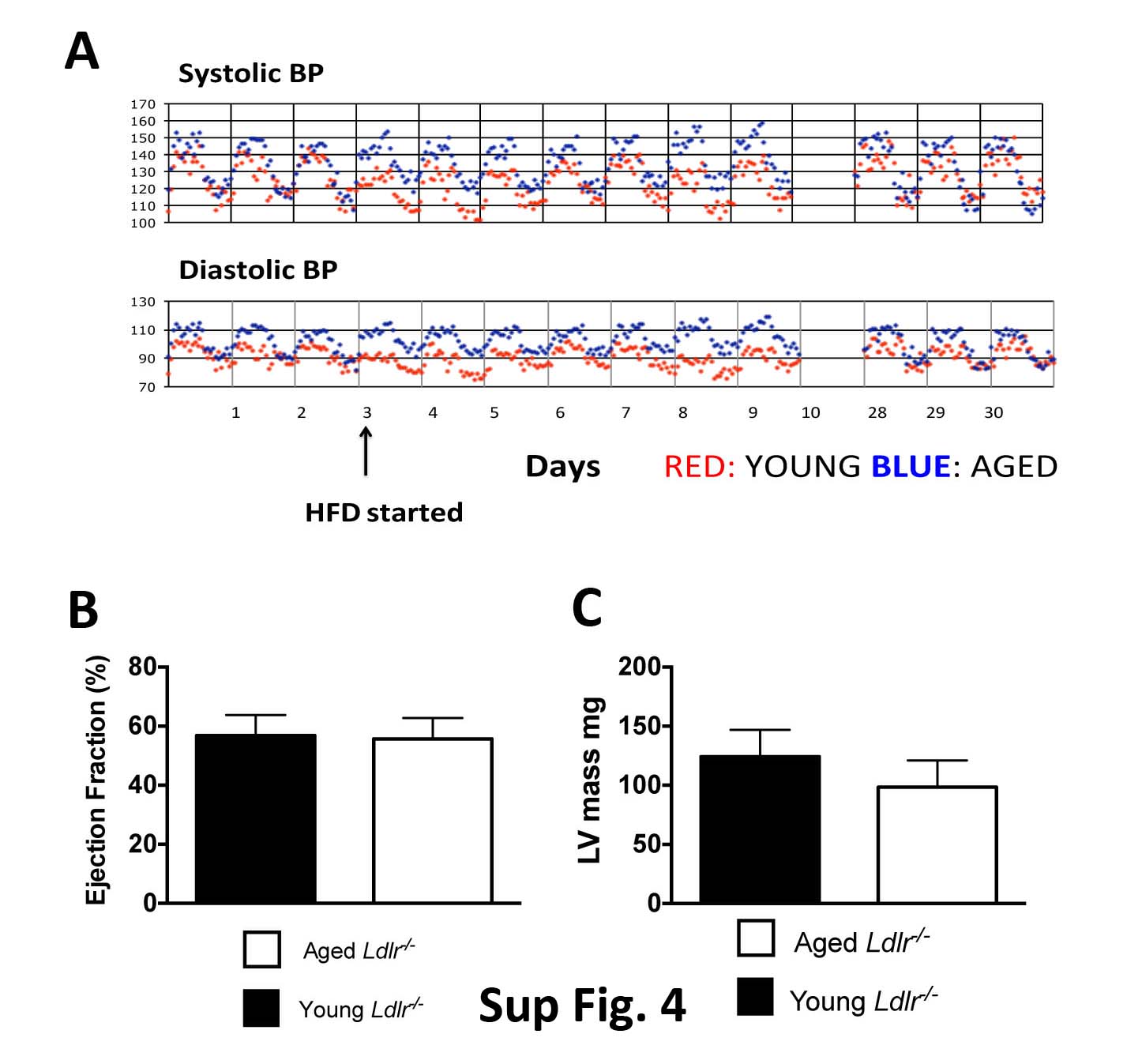

Supplement: Supplementary file 4 — Fig. S4 Hemodynamic parameters [file ACEL-15-766-s004.jpg]

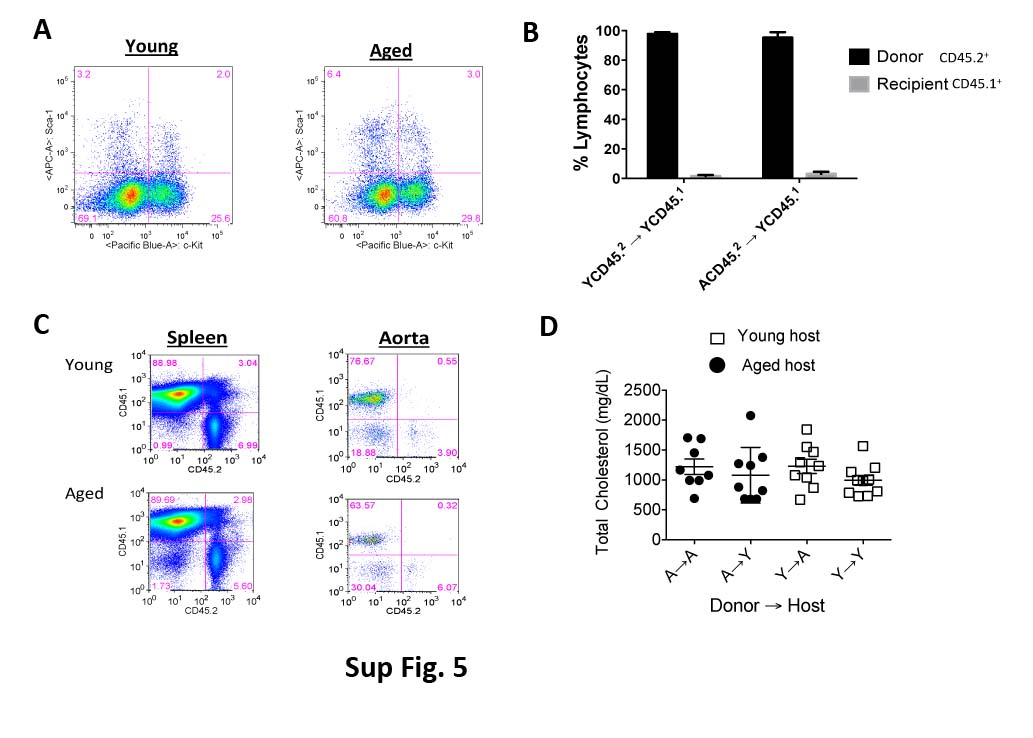

Supplement: Supplementary file 5 — Fig. S5 Bone marrow transplant parameters [file ACEL-15-766-s005.jpg]

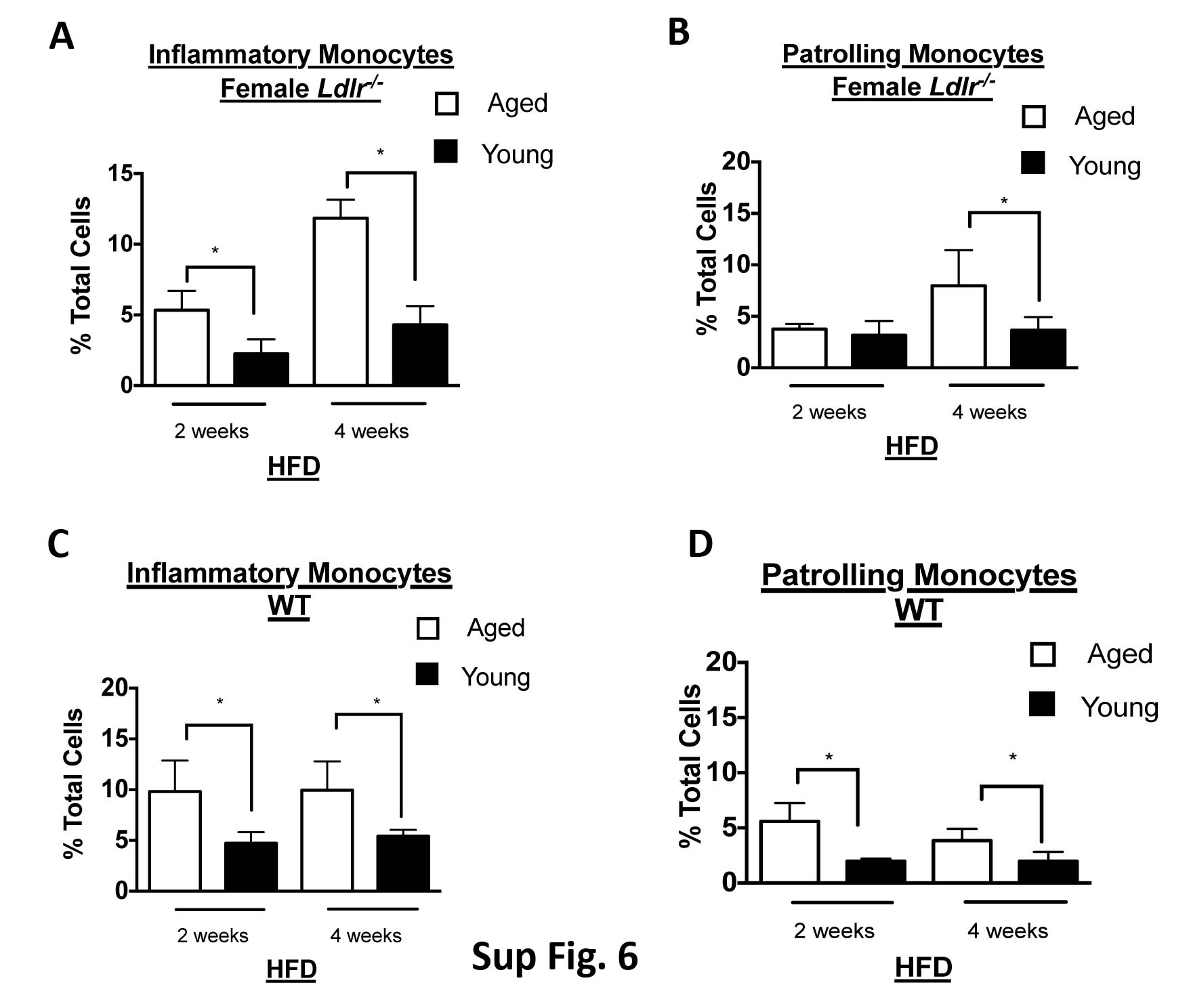

Supplement: Supplementary file 6 — Fig. S6 Inflammatory monocytosis in young and aged female Ldlr −/− mice, and in young and aged WT mice [file ACEL-15-766-s006.jpg]

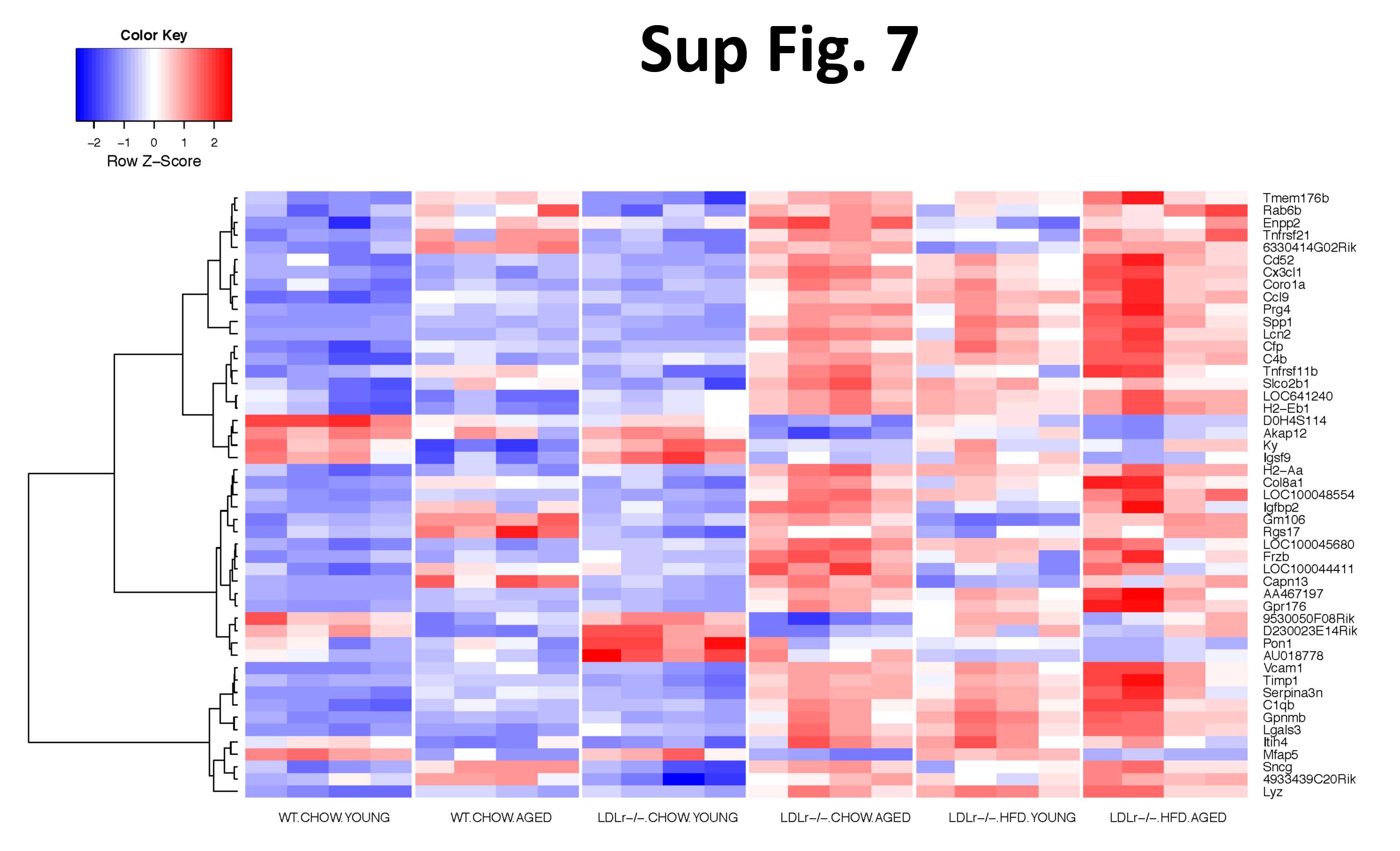

Supplement: Supplementary file 7 — Fig. S7 Heatmap of differentially regulated gene between aortas of young and aged mice. [file ACEL-15-766-s007.jpg]

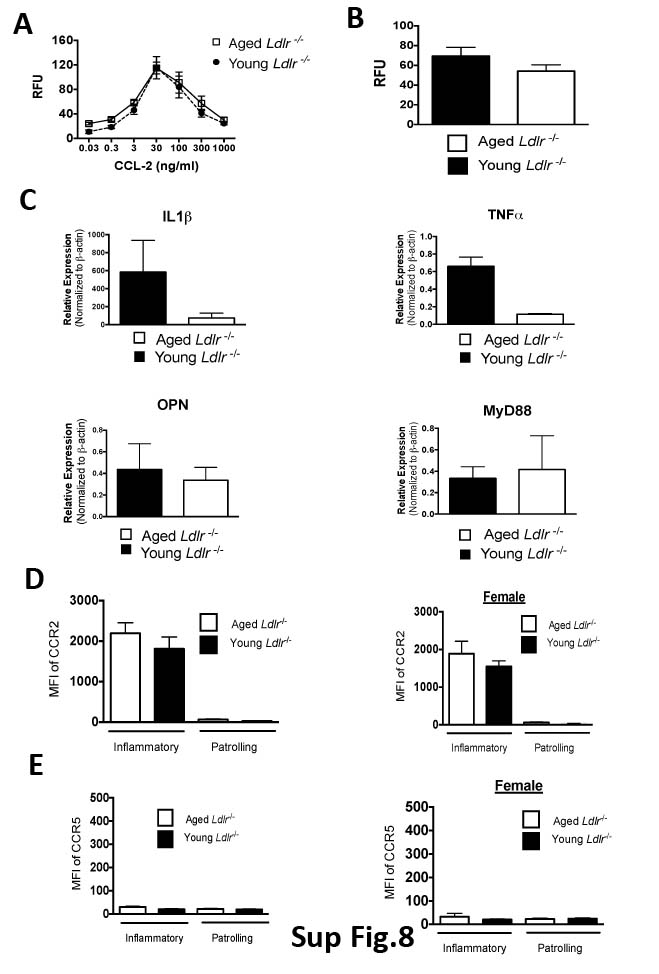

Supplement: Supplementary file 8 — Fig. S8 Impact of aging on monocyte chemotaxis and basal inflammatory responses. [file ACEL-15-766-s008.jpg]

**Rebuttal Figures a and b**  
*For review purposes only*

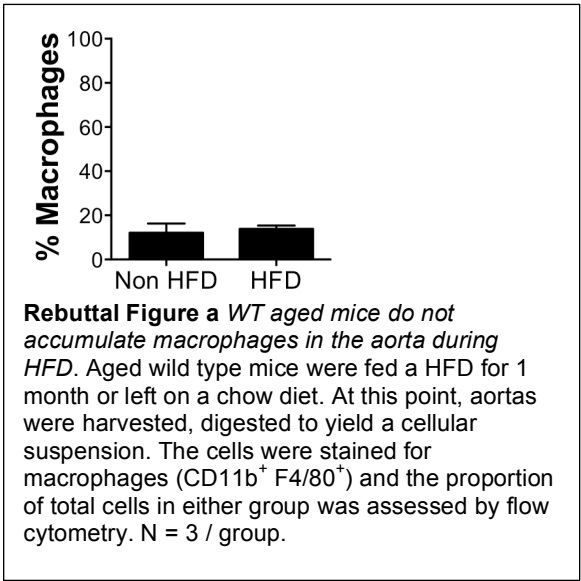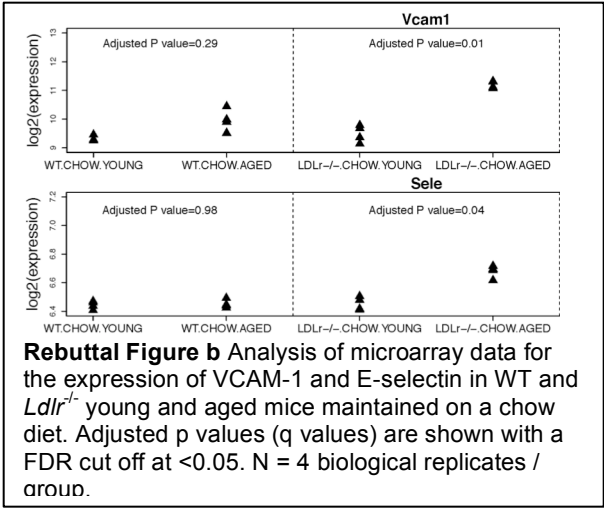

Supplement: Supplementary file 9 [file ACEL-15-766-s009.pdf]
