# Supplementary material for: Age‐associated vascular inflammation promotes monocytosis during atherogenesis
Source: Aging Cell. 2016 May 2;15(4):766–77. doi: 10.1111/acel.12488 (PMC4933655; doi:10.1111/acel.12488)
Supplement: Supplementary file 10 — Data S1. Material and Methods. Table S1 Lists of genes that were significantly down or upregulated in WT young vs. aged mice on a chow diet, young vs. aged Ldlr −/− mice on a chow diet; and young vs. aged Ldlr −/− mice fed a HFD for 3 months. Table S2 Upregulated gene sets between the aortas of aged and young Ldlr −/− in mice maintained on a chow fed diet. Table S3 Downregulated gene sets between the aortas of aged and young Ldlr −/− in mice maintained on a chow‐fed diet. [file ACEL-15-766-s010.docx]

**Supplemental Material and Methods**

*Adipose tissue culture and morphometry*: Adipose tissue (peri gonadal and subcutaneous adipose) were harvested, weighed and then cut into small pieces. The tissue was cultured in 2ml DMEM (low glucose content) containing 10% FBS for 12h. Cytokines and chemokines were measured in the culture supernatants via ELISA.

Adipose tissue morphometry:  Peri-gonadal tissue was fixed in 10% formalin and paraffin embedded. Three representative images were obtained per section, for a total of 6 tissue sections/mouse. Four mice / experimental group were randomly selected and analyzed by taking images using a Zeiss Plan Apochromat 20x objective. Adipocyte area and number were obtained using the ImageJ software (NIH, Bethesda, MD).

*Bone marrow transplantation*

*Ldlr* ^-/-^ mice were irradiated with 10 Gray and then infused via i.v. tail vein injection with 1x10^7^ bone marrow cells from aged matched or mismatched *Ldlr* ^-/-^ mice. Mice were rested for 2 months during which they were fed a HFD. After the 2 month period, mice were euthanized and aortic roots were obtained and lesion sizes were assessed as by H and E staining. To determine if an aged *Ldlr* ^-/-^ environment impacted the ability of bone marrow cells to engraft into the aorta and lymphoid organs, young (4 month of age) and aged (13 months of age) *Ldlr*^-/-^ male mice (all CD45.2^+^) were irradiated and infused with CD45.1^+^ WT bone marrow cells from young male mice. 2 months after bone marrow transplantation, the level of CD45.1^+^ cells engraftment into the lymphoid organs or aorta was measured via flow cytometry. To determine if aged bone marrow cells exhibited a differential ability to engraft into lymphoid organs vs. young bone marrow cells, the bone marrow cells from WT CD45.2^+^ young (2-4 months) and aged (14 months of age) male mice were infused into young CD45.1^+^ male mice. One month after bone marrow transplantation, spleens were obtained and the degree of engraftment was assessed via flow cytometry

*Microarray*

RNA was extracted from ascending and descending whole aortas (four biological replicates per group) using PicaPure RNA isolation Kit (Invitrogen) and subsequently transcribed into cDNA using EcoDry reagents (Clontech). The microarray was run on MouseWG-6 v2.0 Expression BeadChip (Illumina).

Raw expression data were normalized using the quantile method provided by the lumi package in R/Bioconductor (Du *et al.* 2008). Differentially expressed genes were identified by an absolute fold-change of 1.5 and a statistically-significant change in expression as determined by LIMMA (Smyth 2005) using a Benjamani-Hochberg false discovery rate cutoff of q < 0.05.

Gene Set Enrichment tests were performed using QuSAGE version 1.3.1 (Yaari *et al.* 2013). The gene sets used in the analysis contained KEGG and REACTOME gene sets downloaded from MSigDB database v4.0 (<http://www.broadinstitute.org/gsea/msigdb/collections.jsp#C2>)(Abbas *et al.* 2005; Palmer *et al.* 2006). QuSAGE was run twice on the microarray data. First, the *Ldlr^-/-^*group was compared to its aged-matched WT control independently for young and aged groups to identify a list of candidate gene sets associated with LDLr deficiency (Benjamani-Hochberg false discovery rate cutoff of FDR < 0.05 and absolute pathway activity > 0.1). Then QuSAGE was performed across the age groups on the candidate gene sets selected from previous analysis. This experimental design first accounts for differences between WT and LDLr deficiency within each age group and by then comparing across the age groups, determines the age-impacted gene sets associated with atherosclerosis.  The age-impacted gene sets associated with atherosclerosis were detected by FDR < 0.3.

The microarray data have been deposited in NCBI's Gene Expression Omnibus) (Edgar *et al.* 2002) and are accessible through GEO Series accession number GSE69187 (http://www.ncbi.nlm.nih.gov/geo/query/acc.cgi?acc= GSE69187).

*Blood pressure measurement*

Ambulatory blood pressure was recorded at the George M. O’Brien Kidney renal physiology core at Yale School of Medicine. Specifically, blood pressure transducers were placed into the carotid artery of mice (Data Sciences International). Mice were anesthetized with inhaled isoflurane (1-3% in oxygen) and sterile surgical technique was used. Fur was removed in the neck area via shaving and the area washed, treated with 70% ethanol and betadine. A small incision was made to expose the left carotid artery. The blood pressure transducer catheter was inserted and secured in place with silk suture. A subcutaneous pocket was formed on the right lateral flank by blunt dissection to insert the body of the transducer. The skin was closed with surgical staples or absorbable sutures and the mouse was allowed to recover. The procedure lasted approximately 30-45 minutes. After several days, when the mouse was fully recovered and moving freely about the cage, it was singly housed and placed on a special receiver unit connected to a computer that monitors blood pressure. Post-operative analgesics (ibuprofen, 30 mg/kg/day) were administered for 48 hours in the drinking water. Animals were observed daily for the first 2 days and 2-3 times per week until euthanasia. After blood pressure recordings were stable, BP measurements were obtained either on a chow or HFD.

*Echocardiography*

Echocardiograph was performed on anesthetized mice as previously described (Li *et al.* 2013). Briefly, mice were lightly anesthetized with inhaled 1–2% (vol/vol) isoflurane and constant body temperature was maintained. Anatomic M-mode tracings were acquired from parasternal short-axis images via a portable echocardiography device (VisualSonics 2100) and mass and fractional shortening of the left ventricle was subsequently calculated.

*Gene expression in monocytes by real time PCR*

Bone marrow monocytes were isolated and enriched from young (5 months) and aged (12 months) *Ldlr ^-/-^* maintained on a chow diet using the EasySep™ Mouse Monocyte Enrichment Kit (Stemcell). RNA of purified bone marrow monocytes (>96% purity) was extracted using Trizol (Invitrogen) according to the manufacturer’s protocol. Purified RNA was treated with DNAse for 10 minutes at 37°C followed by reverse transcription to cDNA with EcoDry™ Premix Random Hexamers (Clontech) according to the manufacturer’s protocol. qPCR was performed in duplicates using 50ng cDNA / well in 96 well plates and a BioRad CFX96 Touch™ Real-Time PCR Detection System. Relative quantification with β-actin as a housekeeping gene was determined using the 2^-Δ(ΔCt)^ method.

Primer sequences:

|  | Forward | Reverse |
| --- | --- | --- |
| β-actin | CCGCCCTAGGCACCAGGGTG | GGCTGGGGTGAAGGTCTCAAA |
| IL1β | TGTGAAATGCCACCTTTTGA | GGTCAAAGGTTTGGAAGCAG |
| IL6 | TGATGCACTTGCAGAAAACA | ACCAGAGGAAATTTTCAATAGGC |
| TNFα | CCACCACGCTCTTCTGTCTAC | AGGGTCTGGGCCATAGAACT |
| CCL2 | CCTGCTGTTCACAGTTGCC | ATTGGGATCATCTTGCTGGT |
| MyD88 | ACTGAAGGAGCTGAAGTCGC | CACCTGTAAAGGCTTCTCGG |
| OPN | ATTTGCTTTTGCCTGTTTGG | TGGCTATAGGATCTGGGTGC |

**Supplemental Table 1** Lists of genes that were significantly down or upregulated in WT young vs. aged mice on a chow diet, young vs. aged *Ldlr^-/-^* mice on a chow diet; and young vs. aged *Ldlr^-/-^* mice fed a HFD for 3 months.

**WT aged vs. WT young on chow diet:**

|  | |  | |  | |  | |  |
| --- | --- | --- | --- | --- | --- | --- | --- | --- |
| **Gene Symbol** | | **Prob id** | | **Log 2 fold change** | | **P value** | | **Up/down** |
| 2900055J20Rik | | ILMN_2473521 | | -0.791427535 | | 1.29E-06 | | -1 |
| 3830612M24 | | ILMN_2426480 | | -0.652200295 | | 8.62E-05 | | -1 |
| 5430432N15Rik | | ILMN_2622089 | | -0.74258882 | | 7.93E-06 | | -1 |
| 6720458D17Rik | | ILMN_1242427 | | -0.668415401 | | 1.88E-05 | | -1 |
| A530016E13Rik | | ILMN_1232222 | | -0.664678758 | | 3.39E-06 | | -1 |
| Aoc3 | | ILMN_2625920 | | -0.747803527 | | 1.34E-06 | | -1 |
| Cercam | | ILMN_2671755 | | -0.864199273 | | 1.32E-06 | | -1 |
| Cirbp | | ILMN_2761594 | | -0.863210225 | | 1.03E-05 | | -1 |
| Col15a1 | | ILMN_1222111 | | -0.897882628 | | 6.41E-05 | | -1 |
| Col1a1 | | ILMN_2687872 | | -1.611204213 | | 1.26E-06 | | -1 |
| Col1a2 | | ILMN_1253806 | | -0.83083945 | | 1.65E-05 | | -1 |
| Col5a1 | | ILMN_2748402 | | -0.623926668 | | 1.76E-06 | | -1 |
| Col6a1 | | ILMN_2768087 | | -0.804405351 | | 3.77E-07 | | -1 |
| Col6a2 | | ILMN_1216661 | | -0.932460305 | | 9.97E-05 | | -1 |
| D0H4S114 | | ILMN_2680054 | | -1.136157509 | | 6.26E-07 | | -1 |
| D230023E14Rik | | ILMN_2582302 | | -0.595167397 | | 0.000102743 | | -1 |
| Dbp | | ILMN_2616226 | | -0.675477727 | | 0.000312617 | | -1 |
| Eln | | ILMN_2697304 | | -1.096285545 | | 6.26E-05 | | -1 |
| Fbn1 | | ILMN_1223552 | | -0.673509961 | | 9.28E-06 | | -1 |
| Fbxo10 | | ILMN_2529395 | | -0.600541153 | | 6.38E-05 | | -1 |
| Galntl1 | | ILMN_1237583 | | -0.753439158 | | 7.08E-05 | | -1 |
| Hspg2 | | ILMN_2685329 | | -0.737838911 | | 3.34E-06 | | -1 |
| Igsf9 | | ILMN_2804166 | | -0.995698461 | | 4.50E-05 | | -1 |
| Jun | | ILMN_2646625 | | -0.859175431 | | 0.000159421 | | -1 |
| Kit | | ILMN_1246876 | | -0.787782813 | | 0.000140436 | | -1 |
| Knsl5 | | ILMN_1250752 | | -0.965354592 | | 0.000551156 | | -1 |
| Ky | | ILMN_2906855 | | -1.232872341 | | 1.55E-06 | | -1 |
| Lgr6 | | ILMN_2609504 | | -0.595730016 | | 0.000141015 | | -1 |
| LOC100047427 | | ILMN_1229091 | | -0.896981961 | | 0.000186604 | | -1 |
| LOC670044 | | ILMN_2773835 | | -0.733530802 | | 0.000236414 | | -1 |
| Loxl1 | | ILMN_1255871 | | -0.671330081 | | 4.77E-06 | | -1 |
| Ltbp3 | | ILMN_2776619 | | -0.606840084 | | 1.75E-05 | | -1 |
| Med25 | | ILMN_2804622 | | -0.601030369 | | 0.000265341 | | -1 |
| Mfap5 | | ILMN_1225835 | | -0.87356003 | | 2.24E-07 | | -1 |
| Mmp17 | | ILMN_2773800 | | -0.633018485 | | 0.001148251 | | -1 |
| Myh10 | | ILMN_1226114 | | -0.674813154 | | 0.000143016 | | -1 |
| Nfic | | ILMN_1218384 | | -0.656267835 | | 0.000158686 | | -1 |
| Nr1d1 | | ILMN_2749669 | | -0.752607057 | | 0.000186851 | | -1 |
| Nrm | | ILMN_2733524 | | -0.598967114 | | 5.21E-06 | | -1 |
| Nucb1 | | ILMN_2639809 | | -0.604089643 | | 0.00020103 | | -1 |
| Pcolce2 | | ILMN_2678421 | | -0.845940755 | | 0.000292947 | | -1 |
| Pdgfb | | ILMN_2618714 | | -0.626061262 | | 8.13E-05 | | -1 |
| Rrad | | ILMN_1219106 | | -1.004773361 | | 1.22E-05 | | -1 |
| Scube3 | | ILMN_2780286 | | -0.760699459 | | 1.01E-05 | | -1 |
| Slc6a9 | | ILMN_2667384 | | -0.631347413 | | 7.16E-05 | | -1 |
| Sparc | | ILMN_3136561 | | -0.703814025 | | 3.56E-06 | | -1 |
| Tceal5 | | ILMN_2630772 | | -0.617691086 | | 1.37E-07 | | -1 |
| Tmem86a | | ILMN_2645662 | | -0.865302433 | | 9.13E-05 | | -1 |
| Tnxb | | ILMN_2440823 | | -0.854718621 | | 0.000135238 | | -1 |
| Ttc28 | | ILMN_1220397 | | -0.627035586 | | 1.31E-05 | | -1 |
| Vstm2b | | ILMN_1251276 | | -0.696342258 | | 0.000109661 | | -1 |
| Wdr6 | | ILMN_3162081 | | -0.684162745 | | 2.92E-06 | | -1 |
| 1200016E24Rik | | ILMN_2476733 | | 1.25123204 | | 0.00038072 | | 1 |
| 2300002D11Rik | | ILMN_3119914 | | 1.221170669 | | 7.15E-09 | | 1 |
| 4732473B16Rik | | ILMN_2724469 | | 0.599629663 | | 0.00052714 | | 1 |
| 4930544G21Rik | | ILMN_2841307 | | 1.857739604 | | 4.95E-07 | | 1 |
| 6330405H19 | | ILMN_2731908 | | 0.674590541 | | 4.96E-10 | | 1 |
| 6330414G02Rik | | ILMN_1250956 | | 1.711050396 | | 4.26E-11 | | 1 |
| AA407270 | | ILMN_2913989 | | 0.631727719 | | 7.80E-07 | | 1 |
| Ahsg | | ILMN_2764036 | | 0.600913712 | | 0.000558553 | | 1 |
| AI607873 | | ILMN_1254577 | | 0.830589426 | | 0.001041544 | | 1 |
| Art3 | | ILMN_1223147 | | 0.696433528 | | 0.000917394 | | 1 |
| Capn13 | | ILMN_3160190 | | 0.871570531 | | 2.91E-07 | | 1 |
| Ccl9 | | ILMN_2776603 | | 0.94808621 | | 2.16E-05 | | 1 |
| Chordc1 | | ILMN_1247694 | | 0.85545564 | | 0.000216387 | | 1 |
| Cp | | ILMN_2520239 | | 0.587178667 | | 0.000132776 | | 1 |
| Ddit4 | | ILMN_2993109 | | 0.895724686 | | 0.000417343 | | 1 |
| Dpep1 | | ILMN_2754551 | | 0.878080594 | | 1.69E-05 | | 1 |
| Enpp2 | | ILMN_2954474 | | 0.672432979 | | 2.21E-05 | | 1 |
| Errfi1 | | ILMN_2714031 | | 0.818611668 | | 0.001032522 | | 1 |
| F13a1 | | ILMN_2914938 | | 0.765081086 | | 7.47E-06 | | 1 |
| Fcna | | ILMN_2718589 | | 1.069368861 | | 3.98E-05 | | 1 |
| Gabarapl1 | | ILMN_1236958 | | 0.830396268 | | 3.06E-08 | | 1 |
| Gm106 | | ILMN_1244310 | | 1.114188161 | | 2.92E-10 | | 1 |
| Hsd11b1 | | ILMN_3115917 | | 0.817131787 | | 3.71E-06 | | 1 |
| Hsp105 | | ILMN_2716098 | | 1.153782763 | | 0.000378116 | | 1 |
| Hspa8 | | ILMN_1246458 | | 0.656380058 | | 0.000290374 | | 1 |
| Il33 | | ILMN_1259747 | | 0.706796759 | | 4.89E-06 | | 1 |
| Lgi3 | | ILMN_2994995 | | 0.695411523 | | 0.000432347 | | 1 |
| LOC100047583 | | ILMN_2668927 | | 1.77182181 | | 1.12E-08 | | 1 |
| LOC638301 | | ILMN_1257771 | | 0.605493053 | | 0.000192605 | | 1 |
| LOC668631 | | ILMN_1225291 | | 1.099515354 | | 4.15E-09 | | 1 |
| Ly6a | | ILMN_1255416 | | 0.591388588 | | 6.60E-05 | | 1 |
| Man2a1 | | ILMN_1239578 | | 0.599649677 | | 3.73E-06 | | 1 |
| Mmp3 | | ILMN_2753809 | | 1.198467064 | | 6.74E-05 | | 1 |
| Mpp6 | | ILMN_3007680 | | 0.700343694 | | 0.000126837 | | 1 |
| Npy1r | | ILMN_1259965 | | 0.645905354 | | 0.000211502 | | 1 |
| Nxph1 | | ILMN_2659896 | | 0.701776412 | | 8.65E-05 | | 1 |
| Osmr | | ILMN_2623280 | | 0.892314089 | | 1.17E-05 | | 1 |
| Rasl10b | | ILMN_2468981 | | 0.692222101 | | 2.86E-05 | | 1 |
| Rgs17 | | ILMN_1219289 | | 1.153776151 | | 8.20E-07 | | 1 |
| Rhou | | ILMN_1237773 | | 0.635223737 | | 0.000281121 | | 1 |
| Rprd1a | | ILMN_1239180 | | 0.699724114 | | 2.74E-05 | | 1 |
| Sepp1 | | ILMN_3141048 | | 0.621414192 | | 0.000639464 | | 1 |
| Serinc2 | | ILMN_2826264 | | 0.595641266 | | 3.21E-10 | | 1 |
| Sncg | | ILMN_2598478 | | 0.930766347 | | 5.23E-06 | | 1 |
| Sult1a1 | | ILMN_2745370 | | 0.717042515 | | 0.000202362 | | 1 |
| Tmem176b | | ILMN_1259470 | | 0.633419231 | | 0.000111515 | | 1 |
| Tnfrsf21 | | ILMN_2901626 | | 0.813969346 | | 0.000265577 | | 1 |
| Tnfrsf25 | | ILMN_2510383 | | 0.695501 | | 0.001081694 | | 1 |
| Tsc22d3 | | ILMN_3150811 | | 1.011547674 | | 6.04E-05 | | 1 |
| Upk3b | | ILMN_2941714 | | 1.059191812 | | 2.21E-05 | | 1 |
| Wdr92 | | ILMN_1225370 | | 1.386196715 | | 3.77E-05 | | 1 |
|  |  | |  | |  | |  | |

***Ldlr^-/-^* young vs. *Ldlr^-/-^* aged on chow diet**

| **Gene Symbol** | **Prob id** | **Log 2 fold change** | **P value** | **Up/down** |
| --- | --- | --- | --- | --- |
| AU018778 | ILMN_1238140 | -0.848002497 | 0.000316486 | -1 |
| D0H4S114 | ILMN_2680054 | -0.738068219 | 0.000160261 | -1 |
| D230023E14Rik | ILMN_2582302 | -0.775627708 | 3.77E-06 | -1 |
| Igsf9 | ILMN_2804166 | -0.948596445 | 7.93E-05 | -1 |
| Ky | ILMN_2906855 | -0.798692428 | 0.000318641 | -1 |
| Mfap5 | ILMN_1225835 | -0.877811282 | 2.08E-07 | -1 |
| Pon1 | ILMN_2676379 | -1.065367992 | 0.000240407 | -1 |
| 4933439C20Rik | ILMN_2944601 | 0.870375465 | 1.90E-06 | 1 |
| 6330414G02Rik | ILMN_1250956 | 1.390392792 | 1.68E-09 | 1 |
| AA467197 | ILMN_3160750 | 1.01968456 | 0.000194449 | 1 |
| C1qb | ILMN_2619620 | 1.627386812 | 0.000216883 | 1 |
| C4b | ILMN_3049559 | 0.92999976 | 2.83E-05 | 1 |
| Capn13 | ILMN_3160190 | 0.691500998 | 7.81E-06 | 1 |
| Ccl9 | ILMN_2776603 | 0.966579657 | 1.69E-05 | 1 |
| Cd52 | ILMN_2910934 | 1.185884299 | 0.000217524 | 1 |
| Cfp | ILMN_1228320 | 0.632665615 | 0.000345846 | 1 |
| Col8a1 | ILMN_1255925 | 0.971709785 | 0.000131483 | 1 |
| Coro1a | ILMN_2714796 | 1.158304179 | 0.000201405 | 1 |
| Cx3cl1 | ILMN_2627041 | 1.192683516 | 2.77E-07 | 1 |
| Enpp2 | ILMN_2954474 | 0.685627217 | 1.74E-05 | 1 |
| Frzb | ILMN_2601155 | 0.65595204 | 0.000246805 | 1 |
| Gm106 | ILMN_1244310 | 0.7301508 | 3.14E-07 | 1 |
| Gpnmb | ILMN_2648669 | 2.14766439 | 0.000128423 | 1 |
| Gpr176 | ILMN_2742912 | 0.725152666 | 0.000344871 | 1 |
| H2-Aa | ILMN_2734729 | 0.897535726 | 1.86E-06 | 1 |
| H2-Eb1 | ILMN_1239102 | 1.073809491 | 0.000101435 | 1 |
| Igfbp2 | ILMN_1236788 | 1.58567068 | 7.98E-05 | 1 |
| Itih4 | ILMN_2718431 | 0.613770289 | 0.000176462 | 1 |
| Lcn2 | ILMN_2712075 | 3.528929603 | 1.84E-06 | 1 |
| Lgals3 | ILMN_1223317 | 2.049192043 | 5.12E-05 | 1 |
| LOC100044411 | ILMN_2455771 | 0.59138583 | 0.000322489 | 1 |
| LOC100045680 | ILMN_2606624 | 0.688972627 | 1.01E-05 | 1 |
| LOC100048554 | ILMN_1238886 | 1.585002587 | 3.96E-07 | 1 |
| LOC641240 | ILMN_2607675 | 1.088079939 | 0.000371894 | 1 |
| Lyz | ILMN_2878071 | 1.452006863 | 4.06E-05 | 1 |
| Prg4 | ILMN_2668333 | 1.665466256 | 9.06E-05 | 1 |
| Rab6b | ILMN_2761430 | 0.594747168 | 9.58E-05 | 1 |
| Rgs17 | ILMN_1219289 | 0.739487034 | 0.000225015 | 1 |
| Serpina3n | ILMN_1246800 | 1.939370935 | 2.50E-05 | 1 |
| Slco2b1 | ILMN_2619707 | 0.699177329 | 4.28E-07 | 1 |
| Sncg | ILMN_2598478 | 1.013579635 | 1.61E-06 | 1 |
| Spp1 | ILMN_2690603 | 3.730686388 | 9.95E-07 | 1 |
| Timp1 | ILMN_3103896 | 1.884387379 | 1.22E-05 | 1 |
| Tmem176b | ILMN_1259470 | 0.891431083 | 1.43E-06 | 1 |
| Tnfrsf11b | ILMN_2513826 | 1.594981387 | 2.59E-05 | 1 |
| Tnfrsf21 | ILMN_2901626 | 0.935545122 | 5.71E-05 | 1 |
| Vcam1 | ILMN_2778655 | 1.715571606 | 7.53E-06 | 1 |

***Ldlr^-/-^* aged vs. *Ldlr^-/-^* young on HFD:**

| **Gene Symbol** | **Prob id** | **Log 2 fold change** | **P value** | **Up/down** |
| --- | --- | --- | --- | --- |
| Adra2a | ILMN_1242170 | -0.650742739 | 7.41E-05 | -1 |
| C7 | ILMN_2536590 | -0.692370602 | 0.000220486 | -1 |
| Cited2 | ILMN_2477221 | -0.618679664 | 0.000300305 | -1 |
| Dbp | ILMN_2616226 | -0.712780851 | 0.000178125 | -1 |
| Fam135a | ILMN_2790188 | -0.696877056 | 6.20E-06 | -1 |
| Kcnc4 | ILMN_2771095 | -0.839682712 | 9.18E-06 | -1 |
| Lmcd1 | ILMN_2907540 | -0.592910967 | 1.59E-05 | -1 |
| Lrrc17 | ILMN_2511456 | -1.045135907 | 9.01E-07 | -1 |
| Mfap5 | ILMN_1225835 | -0.684827594 | 7.29E-06 | -1 |
| Net1 | ILMN_3151722 | -0.723535298 | 0.000117667 | -1 |
| Pcdh7 | ILMN_2881857 | -0.63471233 | 1.37E-05 | -1 |
| Per2 | ILMN_2987862 | -0.634441387 | 0.00053086 | -1 |
| Rfx2 | ILMN_1217353 | -1.457099812 | 2.53E-07 | -1 |
| Rhobtb1 | ILMN_2421179 | -0.655430354 | 0.000304864 | -1 |
| Rock1 | ILMN_2742675 | -0.592800287 | 0.000315096 | -1 |
| Rrad | ILMN_1219106 | -1.140024546 | 2.23E-06 | -1 |
| Tppp3 | ILMN_2655929 | -0.781611144 | 0.000159695 | -1 |
| 6330414G02Rik | ILMN_1250956 | 1.22920024 | 1.34E-08 | 1 |
| Cdkn1a | ILMN_2634083 | 0.900014782 | 9.97E-09 | 1 |
| Comp | ILMN_2773395 | 0.701830535 | 0.000504568 | 1 |
| Ddit4 | ILMN_2993109 | 0.907050769 | 0.000368148 | 1 |
| Fkbp5 | ILMN_2718266 | 0.875199954 | 0.000238249 | 1 |
| Fxyd5 | ILMN_2967266 | 0.738895013 | 0.000250095 | 1 |
| Gm106 | ILMN_1244310 | 1.113444396 | 2.96E-10 | 1 |
| Htatip2 | ILMN_2603834 | 0.648206028 | 9.57E-06 | 1 |
| Lgi3 | ILMN_2994995 | 0.761659545 | 0.000168882 | 1 |
| LOC100047583 | ILMN_2668927 | 1.086171899 | 1.55E-05 | 1 |
| LOC100048554 | ILMN_1238886 | 0.889684106 | 0.000523521 | 1 |
| LOC668631 | ILMN_1225291 | 0.932967624 | 6.14E-08 | 1 |
| Pik3r1 | ILMN_3114641 | 0.626480392 | 4.84E-05 | 1 |
| Serinc2 | ILMN_2826264 | 0.618026971 | 1.67E-10 | 1 |
| Sphk1 | ILMN_1232884 | 0.741518293 | 0.000272715 | 1 |
| Srpx2 | ILMN_2698728 | 0.755962202 | 5.96E-06 | 1 |
| Ugt1a10 | ILMN_2493826 | 0.670415944 | 0.000430229 | 1 |

**Supplemental Table 2** Upregulated gene sets between the aortas of aged and young *Ldlr ^-/-^* in mice maintained on a chow fed diet.

| **Pathway** | **Pathway Activity** | **P value** | **Function** |
| --- | --- | --- | --- |
| REACTOME NEF MEDIATED DOWNREGULATION OF MHC CLASS I COMPLEX CELL SURFACE EXPRESSION | 0.12 | 0.007 | Host viral response: down regulation of MHC class I |
| REACTOME PLATELET ADHESION TO EXPOSED COLLAGEN | 0.28 | 0.013 | Platelet adhesion and hemostasis |
| REACTOME DEGRADATION OF THE EXTRACELLULAR MATRIX | 0.17 | 0.021 | Extracellular matrix |

Significantly upregulated gene sets found after analysis of microarray data. All gene sets shown exhibited a false discovery rate of <0.3. Pathway activity represents the difference in the expression of the gene sets after the *Ldlr^-/-^*  group is compared to its aged-matched WT control and then compared across the age groups.

**Supplemental Table 3** Downregulated gene sets between the aortas of aged and young *Ldlr ^-/-^* in mice maintained on a chow-fed diet.

| **Pathway** | **Pathway Activity** | **P value** | **Function** |
| --- | --- | --- | --- |
| REACTOME APOBEC3G MEDIATED RESISTANCE TO HIV1 INFECTION | -0.25 | 0.001 | Host response to viral infection |
| REACTOME E2F ENABLED INHIBITION OF PRE REPLICATION COMPLEX FORMATION | \| -0.12 \| \| --- \| \|  \| | 0.005 | DNA replication |
| KEGG REGULATION OF AUTOPHAGY | -0.11 | 0.016 | Cell biology and function |
| REACTOME SYNTHESIS OF GLYCOSYLPHOSPHATIDYLINOSITOL GPI | -0.10 | 0.021 | Cell surface anchor protein |

Significantly upregulated gene sets found after analysis of microarray data. All gene sets shown exhibited a false discovery rate of <0.3. Pathway activity represents the difference in the expression of the gene sets after the *Ldlr^-/-^*  group is compared to its aged-matched WT control and then compared across the age groups.

**Supplemental Figure Legends**

*Supplemental Figure 1* *Assessment of atherosclerosis in advanced aged Ldlr-/- mice and young and aged WT mice*

**A-B:** Representative histological images are shown for young (5 months of age), middle aged (11 months of age), aged (15 months of age) and advanced aged (21 months of age) male *Ldlr* ^-/-^ mice that were maintained on a chow diet until 3 months prior to tissue harvest when they were switch to a HFD. At the end of the HFD feeding period, the aortic root was obtained, stained by hemotoxyln and eosin (H and E), and lesion size was enumerated. Representative images are shown in A, and quantification is shown in B. * p<0.01 (t-test) young, middle and aged data are the same images as shown in Figure 1.

**C:** As per A, necrotic core lesion assessment based upon the area of acellular staining within the H and E images of the aortic root of the each of the experimental cohorts. * p<0.01 (t-test). Young, middle and aged data are also shown in Figure 1.

**D:** Representative photographs of the ascending aortic arch of aged (15-16 months of age) or young (2 months of age) WT mice fed a HFD for 8 weeks.

**E:** Graph of fasting cholesterol levels measured at weekly intervals. Young and aged WT mice, along with young *Ldlr^-/-^* mice were fed a HFD and fasting plasma cholesterol levels were measured. N = 3-4 mice / group

*Supplemental Figure 2 Weight assessment in Ldlr ^-/-^ mice*

**A:** Body weight measurement in young and aged male *Ldlr ^-/-^* mice during HFD, n = 19 / group.

**B**: Weight of total subcutaneous and visceral fat tissue from *Ldlr ^-/-^* mice one month after a HFD. *p<0.001 (Mann-Whitney).

**C-D:** Plasma was obtained from young and aged male *Ldlr ^-/-^* mice after 1 month HFD and SAA (C) and adiponectin (D) was measured by ELISA. Error bars = SEM.

*Supplemental Figure 3 Assessment of insulin resistance in WT mice fed HFD*

**A:** Body weight measurement in young (2 months of age) and aged (15-16 months of age) WT mice fed a HFD for 6 weeks.

**B:** Fasting plasma insulin was measured by ELISA obtained from young (2 months of age) and aged (15-16 months of age) WT mice fed a HFD for 6 weeks, p = 0.4 (t-test).

**C-D:** WT young (2-4 months of age) and aged (15-16 months of age) mice were fed a HFD for 6 weeks. Insulin tolerance test (insulin given at 0.75u/kg body weight i.p) was performed in aged and young WT mice after a 4h fast. D: shows area under the curve (AUC), p = 0.3 (Mann-Whitney). N = 5-8 mice / group. Error bars = SEM,

*Supplemental Figure 4 Hemodynamic parameters*

Hemodynamic assessment in young and aged *Ldlr ^-/-^* mice before and after HFD.

**A:** Young (2 months of age) and aged (12 months of age) *Ldlr ^-/-^* mice had BP transducers placed in left carotid artery to allow chronic ambulatory BP to be measured, as described in the experimental procedures section. Several days after the mice had been rested after the procedure, BP was measured every minute. Data shown are 3h mean blood pressure each day before and after the initiation of HFD diet. Diastolic and systolic blood pressure is shown. N = 4 mice / group.

**B-C:** Aged and young *Ldlr^-/-^* mice were administered a HFD for 3 months and then their left ventricular (LV) function (ejection fraction and LV mass) was assessed non-invasively by echocardiography. N = 5 mice / group. Error bars = SEM.

*Supplemental Figure 5 Bone marrow transplant parameters*

*Enumeration of bone marrow stem cells in young and aged mice. Aged and young bone marrow cells induce similar degree of lymphoid chimerism after bone marrow transplantation. Aged Ldlr^-/-^* *mice are similarly engraftable after bone marrow transplantation as compared to young Ldlr^-/-^* *mice.*

**A:** Bone marrow was harvested from young (2 months of age) and aged (12 months of age) male *Ldlr^-/-^* mice that were maintained on a chow-fed diet. Cells were stained by appropriate fluorescently labeled monoclonal antibodies and the fluorescence acquired after flow cytometric analysis.

**B:** 1 x10^7^ bone marrow cells from young (2 months of age, n = 5) or aged (14 months of age, n = 5) C57BL/6 WT CD45.2^+^ mice were infused i.v. into lethally irradiated CD45.1^+^ young mice. One month after transplantation, engraftment of donor cells was assessed in the spleen.

**C:** Aged (13 months of age, n = 3) and young (2 months of age, n = 3) *Ldlr ^-/-^* CD45.2^+^ mice were lethally irradiated and infused i.v. with 1x10^7^ bone marrow cells from a young CD45.1^+^ donor (WT) C57BL/6 mice. Two months after bone marrow transplantation, the spleens and aorta were harvested and cellular suspensions was obtained. The cells were stained with CD45.1^+^/CD45.2^+^ fluorescently tagged antibodies and data were acquired via flow cytometry. Within the spleen or aorta, the majority of immune cells (>90%) in either the aged or young host were of donor origin. The proportion of donor cells of the total cellular pool was reduced within the aged aortas due to an increase proportion of non-immune cells (i.e., CD45.1^-^/2^-^).

**D:** Lethally irradiated *Ldlr ^-/-^*  young and aged mice were infused with aged or aged-mismatched bone marrow from *Ldlr ^-/-^*  donor mice, fed a chow diet for 2 months and then a HFD for another two months. After the HFD, fasting plasma cholesterol levels were measured.

*Supplemental Figure 6* *Inflammatory monocytosis in young and aged female Ldlr ^-/-^ mice, and in young and aged WT mice*

**A:** Young (2-4 months) and aged (14-15 months of age) female *Ldlr* ^-/-^ mice were placed on a HFD diet for the indicated time. At weekly intervals, peripheral blood was obtained and peripheral blood mononuclear cells were isolated. The cells were stained with fluorescently labeled monoclonal antibodies to assess inflammatory monocytes (CD115^+^, Ly6c^hi^ cells) or patrolling (CD115^+^, Ly6c^lo^ ) monocytes. N = 8-10 mice group, error bars = SEM, *p<0.001 (t-test)

**B:** Young (2-4 months of age) and aged (15-16 months of age) WT mice were fed a HFD for indicated time and the numbers of inflammatory and patrolling monocytes were assessed by flow cytometry per A. n = 5-8 mice / group, *p<0.001 (t-test)

*Supplemental Figure 7 Heatmap of differentially regulated gene between aortas of young and aged mice.*

Aortas were obtained from young (2 months) and aged (12 months) WT chow-fed mice, and *Ldlr ^-/-^* mice that were either maintained on a chow diet for a further 3 months or switched to a HFD for 3 months. N = 4 mice / group. After ascending and descending aortas were harvested, mRNA was extracted and cDNA was synthesized. A microarray was subsequently performed on an Illumina platform. Differentially regulated genes are displayed in a heatmap.

*Supplemental Figure 8 Impact of aging on monocyte chemotaxis and basal inflammatory responses.*

*Aging does not impair the ability of monocytes to chemotax and does not increase monocyte inflammatory gene expression profile.*

**A-B:** Monocytes were negative enriched form the bone marrow of young (2 months) and aged (15 months) *Ldlr^-/-^* mice and were placed in a Boyden chamber in response to increasing doses of CCL-2 (**A**) or to the conditioned media of aortas of young *Ldlr ^-/-^* mice that were fed a HFD for 1 month (**B**). No significant differences were noted between the age groups. N = 5 / group. Error bars = SEM.

**C**: Monocytes were enriched from the bone marrow of young and aged *Ldlr^-/-^* mice ( n = 3) that were maintained on a chow diet. Gene expressions for the indicated genes were measured by real time PCR.

**D and E**: Young and aged male and female *Ldlr^-/-^* mice were fed a HFD for 2 weeks when peripheral blood was obtained and peripheral blood mononuclear cells were isolated. The cells were stained with fluorescently labeled monoclonal antibodies to assess patrolling monocytes CD115^+^, Ly6c^lo^ cells), inflammatory monocytes (CD115^+^, Ly6c^hi^ cells) and also the expression or CCR2 and CCR5 within each subpopulation of monocytes. Expression of CCR2 and CCR5 are expressed as median fluorescent intensity (arbitrary units).

**References**

Abbas AR, Baldwin D, Ma Y, Ouyang W, Gurney A, Martin F, Fong S, van Lookeren Campagne M, Godowski P, Williams PM, Chan AC, Clark HF (2005). Immune response in silico (IRIS): immune-specific genes identified from a compendium of microarray expression data. *Genes Immun*. **6**, 319-331.

Du P, Kibbe WA, Lin SM (2008). lumi: a pipeline for processing Illumina microarray. *Bioinformatics*. **24**, 1547-1548.

Edgar R, Domrachev M, Lash AE (2002). Gene Expression Omnibus: NCBI gene expression and hybridization array data repository. *Nucleic Acids Research*. **30**, 207-210.

Li J, Qi D, Cheng H, Hu X, Miller EJ, Wu X, Russell KS, Mikush N, Zhang J, Xiao L, Sherwin RS, Young LH (2013). Urocortin 2 autocrine/paracrine and pharmacologic effects to activate AMP-activated protein kinase in the heart. *Proc Nat Acad Sci USA*. **110**, 16133-16138.

Palmer C, Diehn M, Alizadeh AA, Brown PO (2006). Cell-type specific gene expression profiles of leukocytes in human peripheral blood. *BMC Genomics*. **7**, 115-115.

Smyth G (2005). Limma: linear models for microarray data. In *Bioinformatics and Computational Biology Solutions using R and Bioconductor*. (CV Gentleman R, Dudoit S, Irizarry R, Huber W, , ed). New York:: Springer,, pp. 397-420.

Yaari G, Bolen CR, Thakar J, Kleinstein SH (2013). Quantitative set analysis for gene expression: a method to quantify gene set differential expression including gene-gene correlations. *Nucleic Acids Research*. **41**, e170-e170.
